# Divergent copies of the large inverted repeat in the chloroplast genomes of ulvophycean green algae

**DOI:** 10.1038/s41598-017-01144-1

**Published:** 2017-04-20

**Authors:** Monique Turmel, Christian Otis, Claude Lemieux

**Affiliations:** Institut de Biologie Intégrative et des Systèmes, Département de biochimie, de microbiologie et de bio-informatique, Université Laval, Québec (QC), Canada

## Abstract

The chloroplast genomes of many algae and almost all land plants carry two identical copies of a large inverted repeat (IR) sequence that can pair for flip-flop recombination and undergo expansion/contraction. Although the IR has been lost multiple times during the evolution of the green algae, the underlying mechanisms are still largely unknown. A recent comparison of IR-lacking and IR-containing chloroplast genomes of chlorophytes from the Ulvophyceae (Ulotrichales) suggested that differential elimination of genes from the IR copies might lead to IR loss. To gain deeper insights into the evolutionary history of the chloroplast genome in the Ulvophyceae, we analyzed the genomes of *Ignatius tetrasporus* and *Pseudocharacium americanum* (Ignatiales, an order not previously sampled), *Dangemannia microcystis* (Oltmannsiellopsidales), *Pseudoneochloris marina* (Ulvales) and also *Chamaetrichon capsulatum* and *Trichosarcina mucosa* (Ulotrichales). Our comparison of these six chloroplast genomes with those previously reported for nine ulvophyceans revealed unsuspected variability. All newly examined genomes feature an IR, but remarkably, the copies of the IR present in the Ignatiales, *Pseudoneochloris*, and *Chamaetrichon* diverge in sequence, with the tRNA genes from the rRNA operon missing in one IR copy. The implications of this unprecedented finding for the mechanism of IR loss and flip-flop recombination are discussed.

## Introduction

Descendants from cyanobacteria, the chloroplasts (plastids) of photosynthetic eukaryotes possess their own genome that encodes components necessary for replication and expression as well as for diverse functions of the chloroplasts^1, 2^. Chloroplast genes exhibit a predominantly uniparental mode of inheritance and their substitution rates are moderate^3^, thus providing useful markers for phylogenetic investigations. The low complexity and high copy number of organelle genomes greatly facilitate their characterization. Owing to these properties and the advent of next-generation sequencing, the pace at which chloroplast genome sequences are being produced has dramatically increased during the last five years^4^. As of December 2016, 1382 chloroplast genome sequences of green plants were publicly available in the Organelle Genome Resources of the NCBI reference sequence project, the majority of which (1276/1382) are derived from land plants. In addition to clarifying phylogenetic relationships, comparative chloroplast genome studies in green plants (green algae + land plants) have shown that this genome experienced a variety of changes through time, with the green algae exhibiting a much wider range of architectural diversity compared to their land plant homologs^5–13^.

Although much has been learned about chloroplast genome evolution in green plants, several questions still need to be addressed. One of them concerns the loss of the rRNA operon-encoding inverted repeat (IR), a structural feature conserved in many algae^2^ and almost all land plants^3, 14^. The two copies of the IR sequence, which varies in size from 6.0 kb (in *Pseudendoclonium akinetum* ^15^) to 76 kb (in *Pelargonium X hortorum* ^16^) and contains five to 40 genes, divide the genome into two distinct single copy (SC) regions and thus create a quadripartite structure. Considering its presence across all major green plant lineages, the IR has undoubtedly functional importance, with suggested roles in replication initiation^17^, genome stabilization^18^ and gene conservation^18, 19^. The two IR copies undergo frequent intramolecular recombination to produce isomeric forms that differ in the relative orientations of the SC regions^20^. This flip-flop recombination may help to maintain the gene complement of each SC region by reducing illegitimate recombination between opposite SC regions, especially if short dispersed repeats are not abundant^21, 22^. When mutations occur within the IR, sequence homogeneity of the two IR copies is maintained by gene conversion. In a variety of land plants, with the notable exception of *Pelargonium* ^23^, genes in the IR evolve three-fold more slowly than those in the SC regions, supporting the hypothesis that the copy-correction mechanism is biased against new mutations^19, 24–26^.

The IR has also the ability to undergo slight expansion or contraction, a phenomenon called the ebb and flow^27^. Each event of expansion/contraction usually involves no more than a few hundred nucleotides. Despite frequent variations in IR/SC boundaries, the overall quadripartite structure shows a high degree of conservation in land plants, as shown by the retention of similar gene contents in the large and small SC regions (LSC and SSC regions) of different taxa and the absence of the IR in only a few lineages (e.g. gymnosperms, legumes, *Erodium*)^3, 14^. But the situation differs sharply in green algae, especially in the Chlorophyta — the lineage sister to the Streptophyta (charophytes + land plants) — where the quadripartite structure has been remodeled extensively^5, 8, 10, 13, 15, 28, 29^ and the IR has been lost in multiple lineages^6, 8, 11, 13, 30, 31^. IR losses took place at least six times in charophytes^9^, four times in prasinophytes^8^, seven times in the Trebouxiophyceae^13^, twice in the Ulvophyceae^31^ and once in the Chlorophyceae^6^. Currently, there exists no convincing evidence for the creation of an IR *de novo* from an IR-less chloroplast genome.

Although the mechanisms that led to IR loss are still largely unknown, three possible models have been envisioned. First, IR loss might be the ultimate consequence of repeated events of IR contraction; however, no compelling evidence supports this hypothesis. Indeed, all green plant IRs, with a single known exception (the IR of the angiosperm *Monsonia* ^32^), contain the complete set of genes making up the rRNA operon, implying that erosion of the IR appears to be impeded when the IR/SC boundaries reach the 5′ or 3′ ends of this operon. Second, complete excision of one of the IR sequence might occur in a single step through intramolecular recombination between short direct repeats at the endpoints of this IR sequences. Comparative analyses of some IR-lacking chloroplast genomes with their IR-containing relatives are consistent with this mechanism of IR loss (*e*.*g*. in *Coleochaete* ^9^). Third, a more recent study in which gene orders of the IR-lacking genome of the ulvophycean green alga *Gloeotilopsis planctonica* (Ulotrichales) and the IR-containing genome of *Pseudendoclonium akinetum* (Ulotrichales) were compared led to the hypothesis that IR loss might occur through differential elimination of the gene sequences making up the rRNA operon in the two IR copies^31^. Obviously, identifying ulvophycean genomes carrying IR copies with differing gene content would provide strong support for this hypothesis.

In the present study, we analyzed the chloroplast genomes of six additional representatives of the Ulvophyceae, with the goals of gaining deeper insights into the mechanism of IR loss, the variability of the quadripartite structure, the dynamics of intron contents, and the phylogeny of chlorophytes. The freshwater unicellular *Ignatius tetrasporus* and *Pseudocharacium americanum* are members of the Ignatiales, an order that has not previously been sampled for genome analysis, while the other taxa are the marine unicellular flagellate *Dangemannia microcystis* (Oltmannsiellopsidales), the coccoid unicellular *Pseudoneochloris marina* (Ulvales) and the filamentous *Chamaetrichon capsulatum* and *Trichosarcina mucosa* (Ulotrichales). Here, we describe these ulvophycean genomes and compare them with nine previously reported genomes of the Ulvophyceae. We also present the phylogenomic trees we inferred from the chloroplast genome sequences of 100 chlorophytes. Our results have broad implications for the evolution of the IR and the chloroplast genome in general. All six newly examined genomes feature an IR, but remarkably the IR copies of the Ignatiales, *Pseudoneochloris*, and *Chamaetrichon* carry divergent sequences. The consequences of this unprecedented finding for the mechanism of IR loss and the recombinational processes to which the IR is subjected are discussed in this report.

## Results

### General features

**Figure 1:**
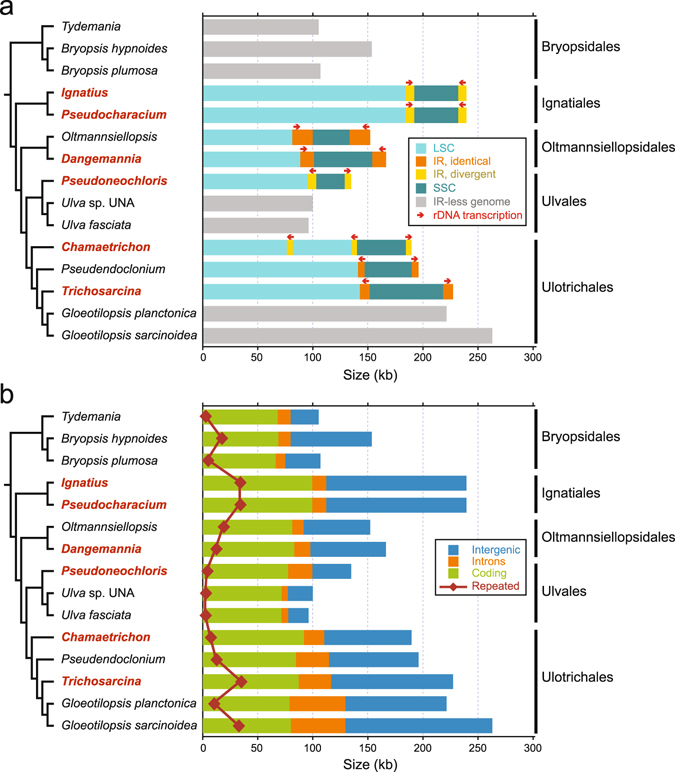
Sequence coverages in the 15 ulvophycean chloroplast genomes compared in this study. (**a**) Sizes of the SSC, IR and LSC regions. Red arrows indicate the direction of transcription of the rRNA operon in IR-containing genomes. Genomes lacking the IR are represented in grey. The names of the newly examined taxa are indicated in red. (**b**) Amounts of coding, intronic, intergenic and small repeated sequences (≥30 bp). Note that intron-encoded genes were not considered as coding sequences but rather as intron sequences. The phylogenetic relationships among the taxa examined are derived from Fig. 2b.

The chloroplast genome sequences of the six newly sampled taxa were assembled as circular-mapping molecules, with sizes ranging from 135 kb (for *Pseudoneochloris*, the representative of the Ulvales) to 239 kb (for the members of the Ignatiales) (Table 1 and Supplementary Figs S1–S6). The summary statistics of these sequence assemblies are reported in Supplementary Table S1 and the general features of the genomes are compared to those of previously examined ulvophyceans in Table 1. All six genomes contain more than one copy of a sequence that encodes the genes making up the rRNA operon; this sequence is designated hereafter as the IR even though the copies differ in both size and sequence in four of the taxa: the two representatives of the Ignatiales (*Ignatius* and *Pseudocharacium*), the ulvalean *Pseudoneochloris* and the ulotrichalean *Chamaetrichon* (Table 1 and Fig. 1a). Remarkably, the latter taxon boasts three copies of the IR, instead of two. Most of the genome size variation observed among ulvophycean taxa can be attributed to variations in intron content and lengths of intergenic and coding regions (Fig. 1b) as well as to differences in IR size (Fig. 1a). The genomes of *Ignatius*, *Pseudocharacium* and the ulotrichalean *Trichosarcina*, which are the largest among the six newly examined taxa, have a moderate number of introns but the highest amount of intergenic sequences (Table 1 and Fig. 1b). Moreover, they exhibit the highest G + C content and the greatest proportion of dispersed repeats ≥30 bp (Table 1 and Fig. 1b).

**Table 1:** General features of ulvophycean chloroplast genomes.

Taxon^a^	Accession	Size (bp)		A + T (%)	Genes^b^ (no.)	Introns (no.)^c^		Repeats^d^ (%)
		Genome	IRA/IRB/IRC			GI	GII	
**Bryopsidales**								
*Tydemania expeditionis* FL1151	NC_026796	105,200	—	67.2	109	8	3	0.4
*Bryopsis hypnoides*	NC_013359	153,426^e^	—	66.9	108^f^	6	6	9.9
*Bryopsis plumosa* West4718	NC_026795	106,859	—	69.2	108	7	6	2.4
**Ignatiales**								
*Ignatius tetrasporus* UTEX 2012*	KY407659	239,387	7,848/7,431	63.0	107	7	2	13.3
*Pseudocharacium americanum* UTEX 2112*	KY407658	239,448	7,848/7,427	63.0	107	7	2	13.3
**Oltmannsiellopsidales**								
*Oltmannsiellopsis viridis* NIES 360	NC_008099	151,933	18,510/18,510	59.5	104	5	0	11.1
*Dangemannia microcystis* SAG 2022*	KY407660	166,355	12,407/12,407	66.3	106	7	1	6.0
**Ulvales**								
*Pseudoneochloris marina* UTEX 1445*	KY407657	134,753	7,524/5,784	70.7	102	7	8	1.4
*Ulva* sp. UNA00071828	KP720616	99,983	—	74.7	100	4	1	0.5
*Ulva fasciata*	NC_029040	96,005	—	75.1	100	4	1	0.5
**Ulotrichales**								
*Chamaetrichon capsulatum* UTEX 1918*	KY407661	189,599	5,189/4,765/5191	69.2	104	15	1	2.7
*Pseudendoclonium akinetum* UTEX 1912	NC_008114	195,867	6,039/6,039	68.5	105	27	0	5.3
*Trichosarcina mucosa* SAG 4.90*	KY407656	227,181	8,979/8,979	62.8	103	7	7	14.4
*Gloeotilopsis planctonica* SAG 29.93	KX306824	221,431	—	68.5	104	14	17	3.7
*Gloeotilopsis sarcinoidea* UTEX 1710	KX306821	262,888	—	68.5	104	15	12	11.6

^a^The taxa newly examined in this study are denoted by asterisks. ^b^Intron-encoded genes and freestanding ORFs not usually found in green plant chloroplast genomes were not considered. Duplicated genes were counted only once. ^c^Numbers of group I (GI) and group II (GII) introns are given. ^d^Nonoverlapping repeat elements were mapped on each genome with RepeatMasker using as input sequences the repeats of at least 30 bp identified with REPuter. ^e^This value is based on the re-annotated version of Leliaert & Lopez-Bautista^35^. ^f^This value includes four genes with frameshift mutations (*rpoB*, *rpoC1*, *ycf20* and *ycf47*).

The genomes of the Ignatiales were found to be the most similar in our study group: they can be aligned over their entire length and differ only at a few sites. This observation raises some doubt about the classification of *Ignatius* and *Pseudocharacium* into separate genera. These two taxa were initially distinguished from each other by the fact that cells of *Pseudocharacium* grow on green algal substrates like *Characium* spp.; however, Watanable and Nakayama^33^ detected no ultrastructural difference between these algae and observed high sequence identity between their 18S rDNAs.

### Phylogenomic analyses

Before comparing the gene content and gene organization of the examined genomes, we present here the phylogenetic context required to interpret these results. Our chloroplast phylogenomic analyses were carried out using amino acid and nucleotide data sets that included 102 green algal taxa (100 chlorophytes and the streptophytes *Mesostigma* and *Chlorokybus*). The amino acid data set (PCG-AA, 14,144 sites) was generated using 79 protein-coding genes, whereas the nucleotide data set (PCG12RNA, 31,893 sites) was assembled from the same set of protein-coding genes (first two codon positions) plus 29 RNA-coding genes (three rRNA and 26 tRNA genes) (see Methods for the gene list). To reduce among-site compositional heterogeneity and thus minimize systematic errors of phylogenetic reconstructions, the most rapidly evolving sites were eliminated from the two data sets. This strategy has recently been reported to produce more robust chloroplast phylogenomic inferences of deep divergences among green algae^34^. The gene locations of the sites that were removed are indicated in Supplementary Fig. S7; they account for 8.4% and 12.8% of the original data from which the PCG-AA and PCG12RNA data sets were derived. Each data set comprises only a small proportion of missing data (8.1% for the PCG-AA and 6.9% for the PCG12RNA data sets).

**Figure 2:**
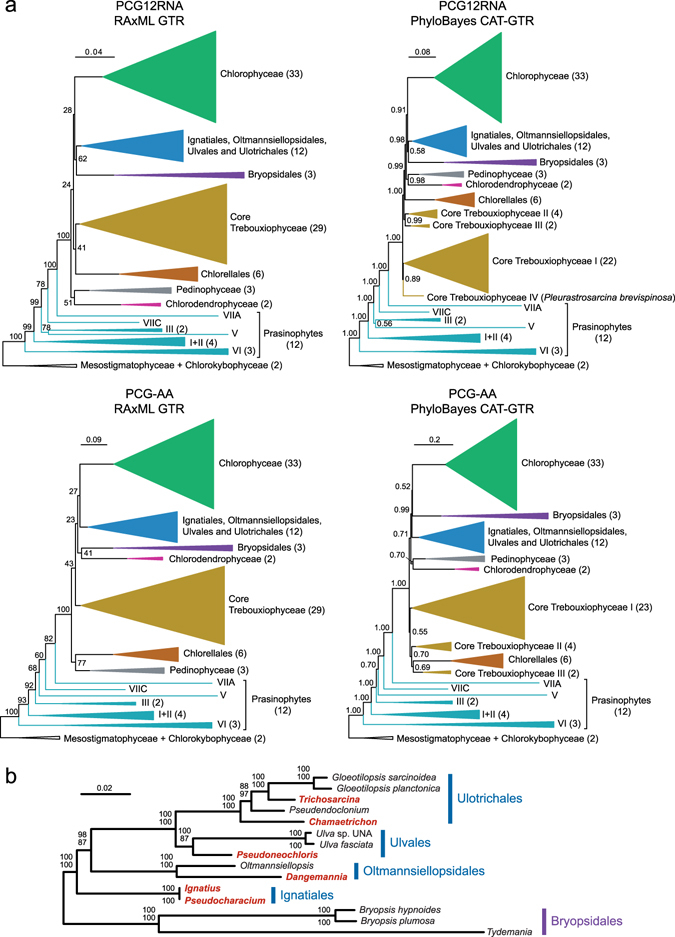
Chloroplast phylogenomic trees of chlorophytes inferred from the PCG-AA and PCG12RNA data sets using RAxML and PhyloBayes. (**a**) Relationships among the major lineages of the Chlorophyta. Strongly supported clades are represented as triangles with sizes proportional to the number of taxa (indicated in parentheses). Bootstrap support and posterior probability values are reported on the nodes. (**b**) Relationships among ulvophycean taxa. The best-scoring RAxML tree inferred from the PCG12RNA data set is presented. Bootstrap support values are reported on the nodes: from top to bottom are shown the values for the RAxML analyses of the PCG12RNA and PCG-AA data sets. The names of the newly examined taxa are indicated in red.

The inferred topologies were dependent upon the data set and the method of analysis, differing mainly with respect to the relative positions of the major lineages in the core Chlorophyta, i.e. the clade sister to prasinophyte lineage VIIA (Fig. 2a and Supplementary Figs S8–S11). Analyses of the PCG12RNA data set using RAxML and PhyloBayes revealed that the class Ulvophyceae is sister to the Chlorophyceae, albeit with no statistical support. In contrast, both the RAxML and PhyloBayes analyses of the PCG-AA data set identified the Ulvophyceae as non-monophyletic, again with no statistical support, with either the Bryopsidales or the clade formed by the Ignatiales, Oltmansiellopsidales, Ulvales, Ulotrichales as sister to the Chlorophyceae. Identical relationships were recovered for the 12 taxa forming the latter clade in the RAxML trees inferred from the two data sets as well as the Bayesian tree inferred from the nucleotide data set (Fig. 2b). In these trees, the Ignatiales was the earliest-diverging lineage, immediately followed by the Oltmannsiellopsidales, and then by the Ulvales and Ulotrichales. The Oltmannsiellopsidales was instead recovered as the earliest divergence in the Bayesian tree inferred from the amino acid data set.

### Gene content

The compared ulvophycean genomes share 95 genes coding for 67 proteins, three rRNAs (*rrs*, *rrl* and *rrf*), and 25 tRNAs (see legend of Supplementary Fig. S12 for the list of common genes). Although *rpoB*, *rpoC1* and *ycf20* sequences were detected in every taxon, these genes were not included in the set of shared genes because frameshift mutations were present in the *Bryopsis hypnoides* genome (Supplementary Fig. S12). Instead of being the consequence of pseudogeneization, these nucleotide changes could be the result of sequencing errors, as such errors were previously detected in other genes of the same genome sequence^35^. From the gene complements of the 15 compared genomes, we predicted that 113 canonical genes were present in the common ancestor of ulvophycean algae and that 15 of them (nine protein-coding genes and six tRNA genes) experienced total loss from the chloroplast in the course of evolution (Supplementary Fig. S12). Seven protein-coding genes (*chlB*, *chlL*, *chlN*, *cysA*, *cysT*, *rpl12* and *ycf47*) were lost only once, with these loss events mapping at two deep nodes and a terminal branch of the ulvophycean phylogeny. The two remaining protein-coding genes (*minD* and *tilS*) were lost on two or three occasions.

Besides canonical genes, we identified freestanding open reading frames (ORFs) showing similarities (*E*-value threshold of 1e-06) with recognized protein domains or previously reported ORFs of unknown function in four of the newly sequenced genomes (Supplementary Table S2). These ORFs encode proteins with domains characteristic of HNH endonucleases, group II intron maturases, reverse transcriptases, and DNA breaking-rejoining enzymes (recombinase/integrase).

### Quadripartite architecture and genome rearrangements

**Figure 3:**
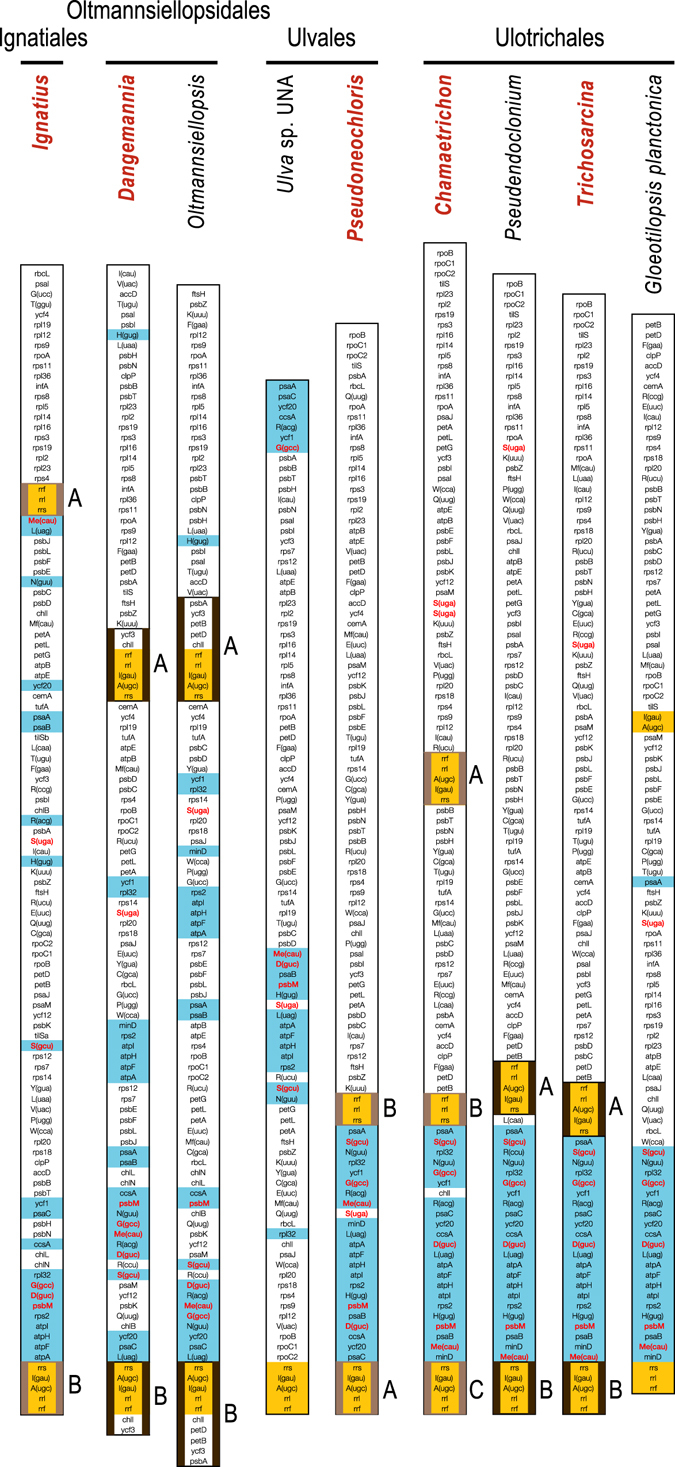
Gene partitioning patterns of ulvophycean chloroplast genomes. The suite of genes in each IR-containing genome is displayed so that the SC region with the gene content the most similar to that predicted for the ancestral SSC region of core chlorophytes is presented at the bottom of the figure. Thick vertical lines delimit the genes encoded in the IR (thick black lines, identical IR copies; thick brown lines, divergent IR copies). The genes making up the rDNA operon are highlighted in yellow whereas those present in the SSC region of *Trichosarcina* are highlighted in blue. Red letterings designate the genes of ancestral LSC origin that have been acquired by the IRs of core chlorophytes.

By comparing the gene contents of the LSC and SSC regions in the investigated ulvophycean genomes, one can observe that the Ignatiales, Oltmannsiellopsidales, and the Ulvales + Ulotrichales exhibit distinct quadripartite architectures (Fig. 3). As previously shown for *Oltmannsiellopsis* ^29^, the longest SC regions in the genomes of *Dangemannia* and the two representatives of the Ignatiales correspond to the SSC region of the ancestral core chlorophyte genome. Although the *Oltmannsiellopsis* and *Dangemannia* LSC regions have exactly the same gene complement, the SSC region of *Dangemannia* differs from its *Oltmannsiellopsis* counterpart by the presence of three extra genes (*psbA*, *petA* and *petB*) which are located in the IR of the latter taxon, thus indicating that the IR underwent contraction/expansion towards the SSC in the Oltmannsiellopsidales. The IRs of the Oltmannsiellopsidales are currently the only known ulvophycean IRs housing protein-coding genes. The IR-containing genomes of the Ulvales and Ulotrichales most closely resemble the ancestral core chlorophyte genome in term of gene partitioning pattern: for instance, only seven of the 23 genes (*psaA*, the *atpA*-*atpF*-*atpH*-*atpI*-*rps2* cluster, and *psaB*) in the SSC region of the ulvalean *Pseudoneochloris* are missing from the SSC of the core chlorophyte ancestor. In the ulotrichalean *Chamaetrichon*, the IRB and IRC copies reside at the same locations as the IR sequences in *Pseudoneochloris* and other ulotrichaleans, while IRA is inserted between *psbB* and *trnR*(ucu), two genes forming a conserved pair in the Ulvales and Ulotrichales. With regards to the IR-lacking genomes from the latter lineages, we note that the gene partitioning pattern characteristic of the IR-containing genomes has been more highly preserved in the Ulotrichales than in the Ulvales.

**Figure 4:**
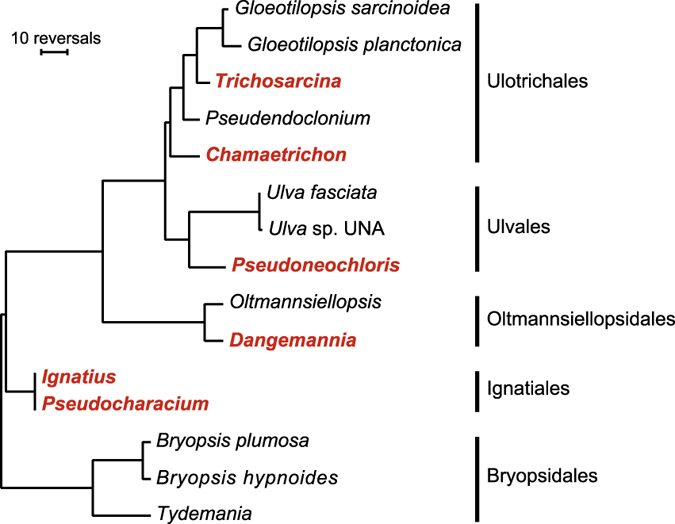
Rearrangements of gene order among ulvophycean chloroplast genomes. The numbers of gene reversals were estimated using MGR v2.03 and the tree topology shown in Fig. 2b. The gene order data set contained 98 genes and included three conserved genes carrying frameshift mutations in *Bryopsis hypnoides* (*rpoB*, *rpoC1* and *ycf20*). The names of the newly examined taxa are indicated in red.

To compare the overall gene organization among ulvophyceans chloroplast genomes, we estimated, using MGR v2.03^36^ and a data set of 98 genes, the numbers of reversals that would be required to interconvert gene order in all possible pairs of genomes. The resulting reversal distances were used to construct the gene rearrangement tree shown in Fig. 4, which is based on the ulvophycean phylogeny reported in Fig. 2b. The results revealed that the chloroplast genomes of the Ignatiales are the least rearranged relative to those of the Bryopsidales. Consistent with the results reported above, the IR-containing genomes sharing the same quadripartite architecture are the most similar in overall gene order. Moreover, gene order is more conserved between IR-lacking and IR-containing genomes in the Ulotrichales than in the Ulvales.

### Divergent IR copies and their Influence on flip-flop recombination

**Figure 5:**
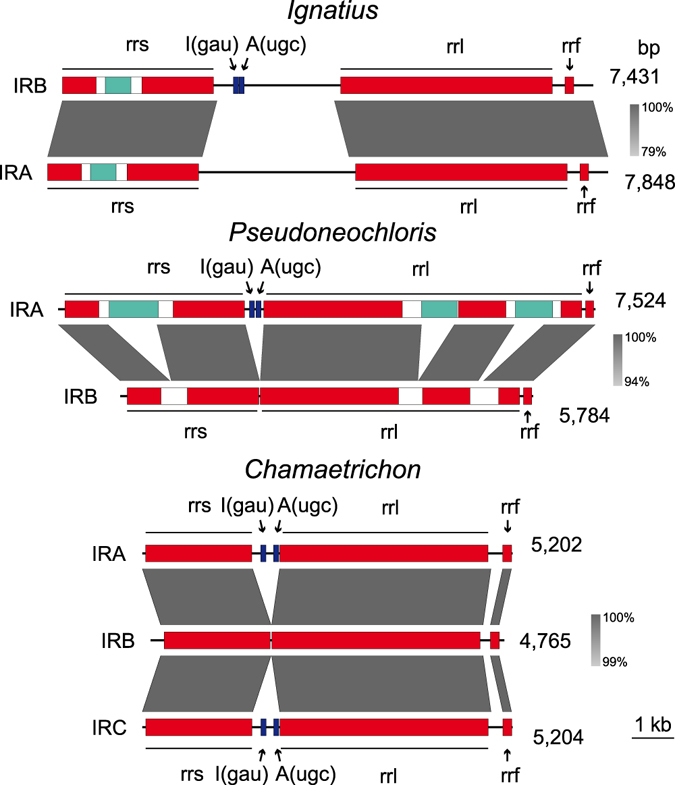
Comparison of IR sequences in the ulvophycean chloroplast genomes carrying non-identical IR copies. Regions displaying similar sequences are connected by shaded areas, with sequence identity denoted by the grey scale. Red and dark blue boxes represent coding regions of rRNA and tRNA genes, respectively; turquoise and white boxes represent ORFs and noncoding regions within introns, respectively.

Among the newly sequenced ulvophycean genomes, identical IR copies are found only in the Oltmannsiellopsidales and the ulvalean *Trichosarcina*. The ulvophycean genomes carrying non-identical IR copies, i.e. those of the Ignatiales, *Pseudoneochloris* and *Chamaetricon*, are all missing *trnI*(gau) and *trnA*(ugc) in one of their IR copies (Figs 3 and 5). IR sequence divergence, however, is not limited to deletion of these tRNA genes. In the Ignatiales, the entire region between *rrs* and *rrl* in the IR copy devoid of tRNA genes (IRA) lacks sequence similarity with the corresponding region in the other IR copy (IRB). Moreover, these regions are substantially larger than their homologs in most other algal chloroplast rRNA operons. In the ulvalean *Pseudoneochloris*, the IR copy with the deleted tRNA genes (IRB) lacks the three intron ORFs present in *rrs* and *rrl*; in addition, the *rrs*/*rrl* spacer of 7 bp in the same copy reflects the deletion of a non-coding sequence relative to the corresponding region in IRA. In the ulotrichalean *Chamaetrichon*, the IR copy missing the tRNA genes (IRB) has clearly lost sequences in the intergenic regions surrounding these genes and the *rrl*/*rrf* spacer has diverged considerably from the corresponding sequences in the two other IR copies. Finally, the IR copies exhibit nucleotide differences in the rRNA genes: we detected a single nucleotide polymorphism in the Ignatiales (5′ end of *rrs*), 16 in *Pseudoneochloris* (10 at the 5′end of *rrs* and the others at the edges of the *rrl* exons), and 8 to 21 nucleotide differences in *Chamaetrichon* (IRA and IRB are the most similar, with six polymorphisms located in *rrl*, while the IRA/IRC and IRB/IRC comparisons revealed 17 and 21 polymorphisms, respectively, most occurring in *rrs*).

**Figure 6:**
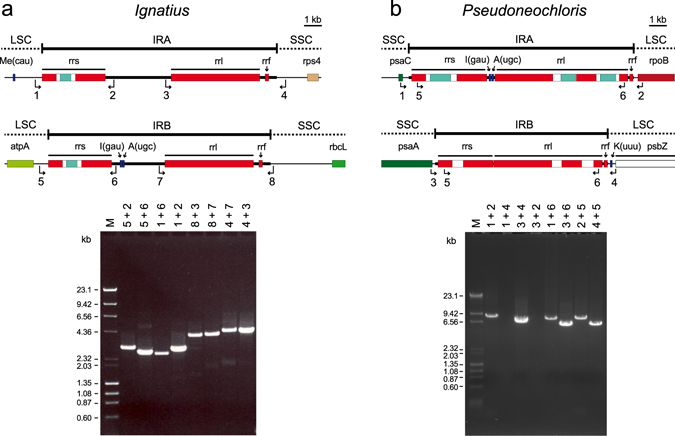
Analysis of flip-flop recombination in the *Ignatius* (**a**) and *Pseudoneochloris* (**b**) chloroplast genomes. PCR assays using eight different pairs of primers were carried out to test whether the non-identical IRs in each algal genome undergo homologous recombination. Primer locations and polarities are indicated by numbered arrows on the diagrams showing the organizations of the IR copies (see Supplementary Table S3 for the primer sequences). PCR products were analyzed by electrophoresis on agarose gels; the numbers above the gel lanes indicate the combinations of primers used.

We undertook a PCR approach to determine whether the divergent IR copies of *Ignatius* and *Pseudoneochloris* participate in flip-flop recombination (Fig. 6). For the PCR assays with *Ignatius*, two primers specific to opposite ends of the SSC region (primers 4 and 8) were used in combination with primers specific to internal sites within the IRA and IRB (primers 3 and 7), while two primers specific to opposite ends of the LSC region (primers 1 and 5) were used in combination with primers specific to IRA and IRB sites (primers 2 or 6). All eight assays yielded products of the expected sizes, indicating that flip-flop recombination occurs in the chloroplast of *Ignatius*. For *Pseudoneochloris*, four PCR assays were designed to generate products that span both the IR/SSC and IR/LSC junctions using combinations of primers specific to genes within the SSC (primers 1 and 3) and LSC (primers 2 and 4) regions. Only two of these essays yielded products, indicating that each IR copy is surrounded by SC regions with a fixed orientation and hence that flip-flop recombination does not take place. Four additional PCR assays, each carried out using a SC-specific primer and a primer complementary to a sequence shared by the IRA and IRB (primers 5 or 6), yielded essentially the same conclusions and confirmed the identities of the genes on either side of the two divergent IR copies.

### Intron distribution

**Figure 7:**
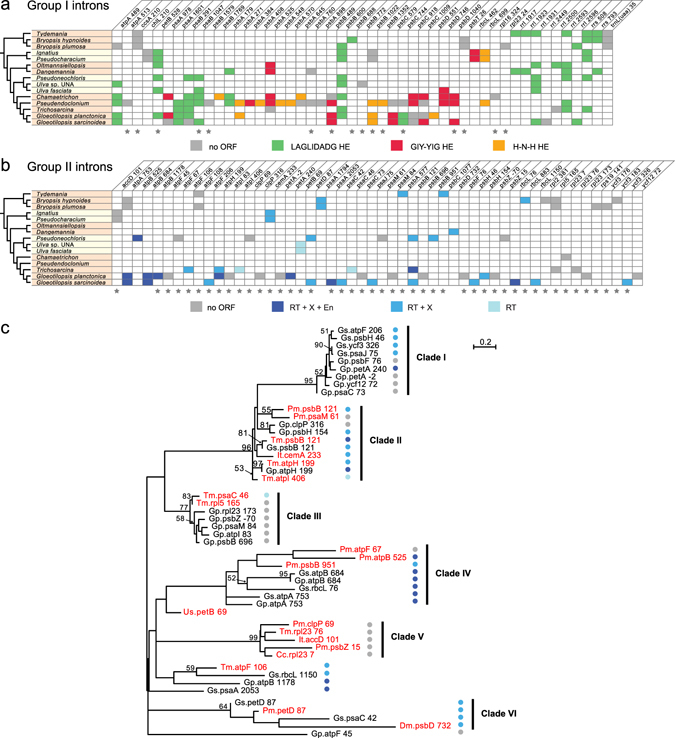
Introns in ulvophycean chloroplast genomes. (**a**) Distribution of group I introns. (**b**) Distribution of group II introns. A grey box denotes an intron lacking an ORF, whereas a colored box represents an intron containing an ORF (see the color code for the type of intron-encoded protein). Stars denote the intron insertion sites that have not been observed in other groups of chlorophytes. Intron insertion sites in protein-coding and tRNA genes are given relative to the corresponding genes in the *Mesostigma* chloroplast genome^67^; insertion sites in *rrs* and *rrl* are given relative to *E*. *coli* 16S and 23S rRNAs, respectively. For each insertion site, the position corresponding to the nucleotide immediately preceding the intron is reported. Abbreviations: EN, H-N-H endonuclease; RT, reverse transcriptase; X, intron maturase. (**c**) Phylogenetic relationships among group II introns of the Ignatiales, Oltmannsiellopsidales, Ulvales and Ulotrichales. The tree shown here was inferred by RAxML analysis of an alignment of 124 nucleotides corresponding to domains IA, IVB, V and VI of the core secondary structure. Bootstrap support values higher than 50% are reported on the nodes. Colored circles denote the types of intron ORF (see the color code on panel b). Note that clades I through IV were identified in a previous phylogenetic study of *Gloeotilopsis* group II introns^31^. Names of the taxa newly examined in the present investigation are indicated in red. Abbreviations: Cc, *Chamaetrichon capsulatum*; Dm, *Dangemannia microcystis*; Gp, *Gloeotilopsis planctonica*; Gs, *Gloeotilopsis sarcinoidea*; It, *Ignatius tetrasporus*; Pm, *Pseudoneochloris marina*; Tm, *Trichosarcina mucosa*; Us, *Ulva* sp. UNA00071828.

All six newly examined ulvophycean taxa have seven group I introns in their chloroplast genome, except *Chamaetrichon* which holds 15 (Table 1). Most of these introns occupy insertion sites that have been previously reported (Fig. 7a). Although two introns in the Ignatiales (*chlL*_210 and *rbcL*_462) and two in *Chaemaetrichon* (*psaB*_1769 and *rpl16*_324) represent novel insertion sites for the Ulvophyceae, only the position of the *Chaemaetrichon rpl16*_324 intron has not been described in other groups of chlorophytes. The intron distribution is irregular, with numerous sites shared between distant lineages of the Ulvophyceae. Homing endonucleases of the LAGLIDADG, GIY-YIG, and HNH families are encoded by ulvophycean group I introns, with the LAGLIDADG genes being the most represented. It is noteworthy that introns sharing a given site always carry the same type of homing endonuclease gene.

Of the six newly examined ulvophyceans, *Pseudoneochloris* and *Trichosarcina* are the only taxa carrying more than two group II introns (Table 1). The 21 group II introns we annotated represent 19 insertion sites (only the ignatialean taxa share introns at the same sites): 15 and 13 of these intron positions have not been previously observed in the Ulvophyceae and chlorophytes, respectively (Fig. 7b). All six ulvophycean taxa contain introns with ORFs; in total, 12 introns encode proteins with reverse transcriptase, intron maturase and/or H-N-H endonuclease domains (Fig. 7b).

To delineate the relationships among the 49 introns currently known in the Ignatiales, Oltmannsiellopsidales, Ulvales and Ulotrichales, a global alignment of 124 nucleotides corresponding to their core secondary structures (domains IA, IVB, V and VI) was submitted to phylogenetic analysis using RAxML under the GTR + G4 model (Fig. 7c). All 21 introns uncovered in the present study, with the exception of the five clustering in clade V (all are ORF-less introns), fall within clades identified in our recent analysis of *Gloeotilopsis* introns^31^. Aside from clades I and III which contain exclusively ulotrichalean introns, all others include members from two or three distinct lineages.

## Discussion

The six newly sequenced IR-containing chloroplast genomes we analyzed in this study highlight the diversity of genome architectures found in the Ulvophyceae. Prior to our investigation, only the IR-containing chloroplast genomes of *Oltmannsiellopsis* ^29^ and of the ulotrichalean *Pseudendoclodium* ^15^ were available for the Ulvophyceae. Early on, it was recognized that their quadripartite structures were distinct from one another and from those of all other chlorophyte genomes that were completely sequenced at the time^29^. In both ulvophycean chloroplast genomes, the SC region encoding the genes usually found in the SSC region of ancestral-type prasinophyte genomes exhibits extra genes, with this SC region being the shortest of the unique regions in *Pseudendoclonium* but the longest in *Oltmannsiellopsis*. Here we report that the ignatialean chloroplast genomes differ substantially in gene partitioning compared to their ulvophycean relatives (Fig. 3). Sampling of a second member of the Oltmannsiellopsidales (*Dangemannia*) revealed that the IR underwent expansion/contraction in this lineage, an event that involved three protein-coding genes but no further modification to the gene contents of the SC regions. In addition, characterization of the first ulvalean IR-containing chloroplast genome (*Pseudoneochloris*) and of two IR-containing chloroplast genomes from the Ulotrichales (*Chamaetrichon* and *Trichosarcina*) disclosed a quadripartite structure identical or highly similar to that of *Pseudendoclonium*. The gene partitioning pattern observed for the Ulvales/Ulotrichales is the closest to that predicted for the common ancestor of all core chlorophytes^10, 13^. As observed for some lineages of the Trebouxiophyceae (*Prasiola* and *Parietochloris* clades)^13^, *psbM* and a set of five tRNA genes (*trnD*(guc), *trnG*(gcc), *trnMe*(cau) *trnS*(gcu), *trnS*(uga)) predicted to have been present in the IR of this ancestor^10^ were transferred to the adjacent SSC region early during the evolution of the Ulvophyceae as a result of IR contraction.

Our finding of divergent IR sequences in the chloroplast genomes of the Ignatiales, *Pseudoneochloris* (Ulvales) and *Chamaetrichon* (Ulotrichales) is an unprecedented observation for the Viridiplantae. In these ulvophycean genomes, one of the IR copies contains all five genes making up the standard rRNA operon, whereas a second copy is missing both *trnI*(gau) and *trnA*(ugc) in the ribosomal intergenic spacer (Fig. 5). The latter copy of the *Pseudoneochloris* IR is also missing three LAGLIDADG endonuclease genes in the group I introns of the *rrs* and *rrl* genes. Non-identical IR copies featuring indels have been previously observed in the chloroplast genomes of haptophytes belonging to the Prymnesiales and Phaeocystales and as reported here for the Ulvophyceae, the genes they encode are restricted to the rRNA operon^37, 38^. Each IR copy of *Chrysochromulina tobin* (Primnesiales) lacks a single tRNA gene (*trnI*(gau) or *trnA*(ugc)) in the ribosomal intergenic spacer^37^, whereas the situation for the IRs of *Phaeocystis antartica* and *Phaeocystis globosa* is identical to what we uncovered in our study, i.e. a standard rRNA operon in one IR copy and only the rRNA genes in the other^38^.

Despite the divergence of the IR sequences, we detected intramolecular recombination between the IR copies of *Ignatius*; however, no isomers were identified for the *Pseudoneochloris* genome (Fig. 6). The absence of flip-flop recombination in the latter genome is correlated with the accumulation of nucleotide polymorphisms in the IR copies. Similar observations (i.e. presence of polymorphisms and absence of recombination) were reported for the haptophyte *Chrysochromulina* ^37^, supporting the view that pairing of the two IR copies for recombination provides a copy-correction mechanism. What triggered the independent losses of the tRNA genes from the IR in the three distinct lineages of the Ulvophyceae? Why were the functional copies of these genes not used as templates for copy-correction of the non-canonical sequences? Was a mutation in a nuclear-encoded gene participating in DNA recombination or DNA repair involved? Even though further investigations are required to answer these questions, it appears that the events linked to the degeneration of the rRNA operon were complex and involved multiple steps.

Aside from the divergent IR copies located at the same positions as in other ulotrichalean chloroplast genomes (copies B and C), the *Chamaetrichon* genome contains a third IR copy (copy A) that is inserted between two genes forming a syntenic pair in the Ulotrichales and Ulvales (Fig. 3). To our knowledge, this is the first time that three copies of the rRNA genes are reported in a chloroplast genome of the Viridiplantae. Although compelling evidence for *de novo* creation of an IR from an IR-less chloroplast genome has not been documented, our finding of a third copy of the rDNA operon in *Chamaetrichon* makes this evolutionary scenario plausible.

The chloroplast IR has been entirely lost multiple times during the evolution of green algae^6, 8, 9, 13, 31^. In the cases of ulvophycean and haptophyte chloroplast genomes carrying short IRs, the process of IR sequence divergence and degeneration of the rRNA operon likely represents an intermediate step towards the complete loss of the IR. But to provide unambiguous evidence for or against this hypothesis, it will be necessary to investigate IR-less genomes from close relatives of taxa carrying divergent IR copies. Note, however, that the data reported here for the Ulotrichales are consistent with our recent comparison of gene order between the *Pseudendoclonium* and *Gloeotilopsis* genomes, which suggested that differential elimination of sequences within the rRNA operon from the two IR copies led to IR loss^31^.

Our study also highlights the diversity of both group I and group II introns in ulvophycean chloroplast genomes. Novel insertion sites were found to be more abundant for the group II introns, especially in the Ulvales and Ulotrichales (Fig. 7). Our phylogenetic analysis of ulvophycean group II introns uncovered a number of clades containing introns originating from different species and insertion sites. This observation suggests that several introns arose by intragenomic proliferation of existing introns, thus echoing our recent conclusions regarding the mobility of group II introns in *Gloeotilopsis* ^31^.

The Ignatiales affiliated with the Oltmansiellopsiales, Ulvales and Ulotrichales to form a strongly supported clade in all phylogenomic trees inferred in this study, but whether the Ignatiales or the Oltmansiellopsidales is the most basal lineage could not be identified unambiguously (Fig. 2). These results are not congruent with the ten-gene phylogenetic analyses of Cocquyt *et al*.^39^, which recovered *Ignatius* with the TBCD (Trentepohliales-Bryopsidales-Cladophorales-Dasycladales) clade, a large assemblage that contains the bulk of the green seaweeds. Previously reported phylogenies based on the nuclear small-subunit rRNA gene^33^ had revealed that *Ignatius* is either embedded in the Ulvales–Ulotrichales clade or clustered with the TBCD clade depending on the inference method used.

Although the major clades of core chlorophytes received high support in all trees we inferred, their precise placements were dependent upon the phylogenetic methods and data sets employed (Fig. 2a). As we pointed out earlier^10^, inference of more robust and reliable trees will probably require a broader sampling of chlorophytes and improved models of sequence evolution. Although the relationships among core chlorophyte lineages remain ambiguous, the newly sequenced ulvophycean genomes reported here strengthen the database of available genomes for future studies aimed at deciphering the phylogenetic relationships among members of specific lineages.

## Materials and Methods

### Strains and culture conditions

*Ignatius tetrasporus* UTEX 2012, *Pseudocharacium americanum* UTEX 2112, *Pseudoneochloris marina* UTEX 1445 and *Chamaetrichon capsulatum* UTEX 1918 were obtained from the culture collection of algae at the University of Texas in Austin, while *Dangemannia microcystis* SAG 2022 and *Trichosarcina mucosa* SAG 4.90 originated from the culture collection at the University of Goettingen. *Ignatius* and *Pseudocharacium* were grown in medium C^40^, while the remaining ulvophyceans were grown in medium K^41^. All cultures were incubated at 18 °C under alternating 12 h-light/12-h dark periods.

### DNA isolation, sequencing and *de novo* assemblies

The chloroplast genomes of *Ignatius*, *Pseudocharacium*, *Dangemannia*, and *Pseudoneochloris* were sequenced using the Roche 454 method, whereas those of *Chamaetricon* and *Trichosarcina* were sequenced using the Illumina method.

For 454 sequencing, A + T-rich organellar DNA was separated from nuclear DNA by CsCl-bisbenzimide isopycnic centrifugation^8^. Shotgun libraries (700-bp fragments) of A + T-rich DNA were constructed using the GS-FLX Titanium Rapid Library Preparation Kit of Roche 454 Life Sciences (Branford, CT, USA). Library construction and 454 GS-FLX DNA Titanium pyrosequencing were carried out by the “Plateforme d’Analyses Génomiques de l’Université Laval” (http://pag.ibis.ulaval.ca/seq/en/). Following trimming of adapter and low-quality sequences with CUTADAPT^42^ and PRINTSEQ^43^, respectively, reads were assembled using Newbler v2.5^44^ with default parameters, and contigs were visualized, linked and edited using CONSED v22^45^. Contigs of chloroplast origin were identified by BlastN and BlastX searches^46^ against a local database of green plant chloroplast genomes. Regions spanning gaps in the assemblies were amplified by polymerase chain reaction (PCR) with primers specific to the flanking sequences. Purified PCR products were sequenced using Sanger chemistry with the PRISM BigDye Terminator Ready Reaction Cycle Sequencing Kit (Applied Biosystems, Foster City, CA, USA) on ABI model 373 or 377 DNA sequencers (Applied Biosystems).

For Illumina sequencing, total cellular DNA was isolated using the EZNA HP Plant Mini Kit of Omega Bio-Tek (Norcross, GA, USA). Libraries of 500-bp fragments were constructed using the TrueSeq DNA Sample Prep Kit (Illumina, San Diego, CA, USA) and paired-end reads were generated on the Illumina HiSeq 2000 (100-bp reads) or the MiSeq (300-bp reads) sequencing platforms by the Innovation Centre of McGill University and Génome Québec (http://gqinnovationcenter.com/index.aspx) and the “Plateforme d’Analyses Génomiques de l’Université Laval”, respectively. Reads were trimmed to remove adapter and low-quality sequences with CUTADAPT^42^ and PRINTSEQ^43^, respectively, and the paired-end sequences were merged using FLASH^47^. The reads were then assembled using Ray v2.3.1^48^ and contigs were visualized, linked and edited using CONSED v22^45^. Identification of chloroplast contigs and gap filling were performed as described above for the 454 sequence assemblies.

### Genome annotations

We used a custom-built suite of bioinformatics tools allowing the automated execution of the following three steps: (1) ORFs were found using GETORF in EMBOSS^49^, (2) their translated products were identified by BlastP searches^46^ against a local database of chloroplast-encoded proteins or the nr database at the National Center for Biotechnology Information, and (3) consecutive 100-bp segments of the genome sequence were analyzed with BlastN and BlastX to determine the approximate positions of RNA-coding genes, introns and exons. Only the ORFs that revealed identities with genes of known functions or previously reported ORFs were annotated. The precise positions of rRNA and tRNA genes were identified using RNAmmer^50^ and tRNAscan-SE^51^, respectively. Intron boundaries were determined by manual modelling of intron secondary structures^52, 53^ and by comparing the sequences of intron-containing genes with those of intronless homologs. Circular genome maps were drawn with OGDraw^54^.

Genome-scale sequence comparisons were carried out with LAST v7.1.4^55^. Comparisons of IR sequences were performed using EasyFig v2.2.2^56^. To estimate the proportion of small repeated sequences, repeats with a minimal size of 30 bp were retrieved using REPFIND of REPuter v2.74^57^ and were masked on the genome sequence using RepeatMasker (http://www.repeatmasker.org/) running under the Crossmatch search engine (http://www.phrap.org/).

### Phylogenomic analyses

The GenBank accession codes used to generate the amino acid and nucleotide data sets (PCG-AA and PCG12RNA, respectively) are provided in Supplementary Table S4. The PCG-AA data set was assembled from 79 protein-coding genes: *accD*, *atpA*, *B*, *E*, *F*, *H*, *I*, *ccsA*, *cemA*, *chlB*, *I*, *L*, *N*, *clpP*, *cysA*, *T*, *ftsH*, *infA*, *minD*, *petA*, *B*, *D*, *G*, *L*, *psaA*, *B*, *C*, *I*, *J*, *M*, *psbA*, *B*, *C*, *D*, *E*, *F*, *H*, *I*, *J*, *K*, *L*, *M*, *N*, *T*, *Z*, *rbcL*, *rpl2*, *5*, *12*, *14*, *16*, *19*, *20*, *23*, *32*, *36*, *rpoA*, *B*, *C1*, *C2*, *rps2*, *3*, *4*, *7*, *8*, *9*, *11*, *12*, *14*, *18*, *19*, *tufA*, *ycf1*, *3*, *4*, *12*, *20*, *47*, *62*. It was prepared as follows: the deduced amino acid sequences from the individual genes were aligned using MUSCLE v3.7^58^, the ambiguously aligned regions in each alignment were removed using TrimAl v1.3^59^ with the options block = 6, gt = 0.7, st = 0.005 and sw = 3, and the protein alignments were concatenated using Phyutility v2.2.6^60^. The 15,447 characters in the concatenated matrix were then sorted into 30 bins according to their rate of variation using TIGER v1.02^61^, and the 1,303 fastest evolving characters identified in bins 29 and 30 were removed from the matrix in an attempt to reduce among-site compositional heterogeneity in the data set.

Phylogenies were inferred from the PCG-AA data set using the maximum likelihood (ML) and Bayesian methods. ML analyses were carried out using RAxML v8.2.6^62^ and the GTR + Γ4 model of sequence evolution; in these analyses, the data set was partitioned by individual gene, with the model applied to each partition. Confidence of branch points was estimated by bootstrap analysis with 100 replicates. Bayesian analyses were performed with PhyloBayes v4.1^63^ using the site-heterogeneous CATGTR + Γ4 model^64^. Five independent chains were run for 2,000 cycles and consensus topologies were calculated from the saved trees using the BPCOMP program of PhyloBayes after a burn-in of 500 cycles. Note that the chains failed to converge under these conditions (maxdiff = 0.86), indicating that at least one of the chains was stuck in a local maximum.

The PCG12RNA data set was prepared from the first and second codon positions of the 79 protein-coding genes abovementioned and from three rRNA and 26 tRNA genes. The multiple sequence alignment of each protein was first converted into a codon alignment, poorly aligned and divergent regions in each codon alignment were excluded using Gblocks v0.91b^65^ with the −t = c, −b3 = 5, −b4 = 5 and −b5 = half options, and the individual gene alignments were concatenated using Phyutility v2.2.6^60^. The third codon positions of the resulting alignment were then excluded using Mesquite v3.04^66^ to produce the PCG12 data set. To obtain the PCG12RNA, the PCG12 matrix was merged with the concatenated alignment of the following RNA genes: *rrf*, *rrl*, *rrs*, *trnA*(ugc), *C*(gca), *D*(guc), *E*(uuc), *F*(gaa), *G*(gcc), *G*(ucc), *H*(gug), *I*(cau), *I*(gau), *K*(uuu), *L*(uaa), *L*(uag), *Me*(cau), *Mf*(cau), *N*(guu), *P*(ugg), *Q*(uug), *R*(acg), *R*(ucu), *S*(gcu), *S*(uga), *T*(ugu), *V*(uac), *W*(cca), *Y*(gua). The latter genes were aligned using MUSCLE 3.7^58^, the ambiguously aligned regions in each alignment were removed using TrimAl v1.3^59^ with the options block = 6, gt = 0.9, st = 0.4 and sw = 3, and the individual alignments were concatenated using Phyutility v2.2.6^60^. The fastest evolving sites in the resulting concatenated alignment of 36,385 nucleotide characters were then identified and removed essentially as described above for the PCG-AA data set. A total of 4,492 characters were eliminated during this step.

ML analysis of the PCG12RNA data set was performed using RAxML v8.2.6 and the GTR + Γ4 model of sequence evolution. The data set was partitioned into gene groups, with the model applied to each partition. The partitions included two RNA gene groups (rRNA and tRNA genes) in addition to the protein-coding gene partitions. Confidence of branch points was estimated by bootstrap analysis with 100 replicates. The Bayesian analyses were performed under the same conditions as those described above for the PCG-AA data set. Here again, the five independent chains failed to converge (maxdiff = 0.78), indicating that at least one of the chains was stuck in a local maximum.

### Analysis of gene rearrangements

A gene reversal tree was inferred using a gene order matrix of 98 genes from 15 ulvophycean chloroplast genomes. The branch lengths of this tree were computed on the tree topology inferred from the RAxML analyses of the sequence data using the -t option of MGR v2.03^36^. Because MGR cannot handle duplicated genes, only one copy of the IR and of each duplicated gene was included in the matrix.

### Phylogenetic analyses of group II introns

Group II intron sequences were aligned manually on the basis of their secondary structure models, and poorly aligned and divergent regions were removed. The data set of 124 sites corresponding to domains IA, IVB, V and VI of the core secondary structure^52^ was analyzed using RAxML v8.2.6^62^ and the GTR + Γ4 model. Confidence of branch points was estimated by bootstrap analysis with 1000 replicates.

### Analyses of chloroplast genome isomers

PCR analyses were performed to test whether the *Ignatius* and *Pseudoneochloris* IRs undergo flip-flop recombination. For each algal IR, multiple pairs of oligonucleotide primers were designed to yield products that overlap all possible boundaries of the IR sequences with the flanking SC regions (see Supplementary Table S3 for the sequences of these primers). PCR assays were carried out using the GeneAmp XL PCR kit (ABI Applied Biosystems, Foster City, CA, USA) and the conditions recommended by the manufacturer.

## Electronic supplementary material

Supplementary Tables and Figures
