# Supplementary material for: Divergent copies of the large inverted repeat in the chloroplast genomes of ulvophycean green algae
Source: Sci Rep. 2017 Apr 20;7:994. doi: 10.1038/s41598-017-01144-1 (PMC5430533; doi:10.1038/s41598-017-01144-1)
Supplement: Supplementary file 1 — Supplementary Tables and Figures [file 41598_2017_1144_MOESM1_ESM.pdf]

Divergent copies of the large inverted repeat in the chloroplast genomes of ulvophycean green algae

(Supplementary Information)

Monique Turmel, Christian Otis, and Claude Lemieux*

Institut de Biologie Intégrative et des Systèmes, Département de biochimie, de microbiologie et de bio-informatique, Université Laval, Québec (QC) Canada

* Corresponding author

E-mail: claude.lemieux@bcm.ulaval.ca

Taxon	Technology	Sequencing reads (no.)		Fold coverage of chloroplast genome sequences						
		Total	cpDNA	Average	IR-SC1	IR-SC2	IR-SC3	IR-SC4	IR-SC5	IR-SC6
Ignatius tetrasporus	Roche 454	49,055	41,372	60	43	32	44	32	–	–
Pseudocharacium americanum	Roche 454	40,038	37,504	58	45	58	43	32	–	–
Dangermannia microcystis	Roche 454	80,710	48,067	108	117	75	36	48	–	–
Pseudoneochloris marina	Roche 454	82,351	59,134	156	161	149	152	113	–	–
Chamaetrichon capsulatum	Illumina	1,997,570	96,903	154	125	105	119	93	173	123
Trichosarcina mucosa	Illumina	4,067,674	275,477	361	320	218	167	323	–	–

Supplementary Table S1. Summary statistics of the chloroplast genome sequencing and assemblies. The sequence coverage values observed at the junctions between IR and single-copy regions (IR-SC1 to IR-SC6) are provided in the order these junctions appear in the GenBank accessions of the chloroplast genomes.

Taxon	ORF[a]	Coordinates[b]	Conserved domain or similar protein
Ignatius tetrasporus	437	22433-21120	HNH endonuclease (pfam01844) with RRXRR domain (pfam14239)
	112	46900-46562	HNH endonuclease with RRXRR domain (pfam14239)
	100	158515-158213	HNH endonuclease
	128	158980-158594	HNH endonuclease (pfam01844)
	106	159328-159008	HNH endonuclease with RRXRR domain (pfam14239)
	98	220328-220624	Transposase
Dangemannia microcystis	246	123637-122897	Group II intron maturase (pfam08388) and HNH (pfam01844) domains
	114	124647-124303	N-terminal domain of reverse transcriptase (pfam13655)
Chamaetrichon capsulatum	680	84264-86306	Hypothetical protein MVEG-06986 of *Mortierella verticillata*
	206	121843-121223	Hypothetical protein PsakCp041 of *Pseudendoclonium akinetum* (*orf239*)
Trichosarcina mucosa	190	67813-68064	DNA breaking-rejoining enzyme, phage integrase family (pfam00589)
	121	86662-87027	Hypothetical protein PsakCp022 of *Pseudendoclonium akinetum* (*orf156*)
	233	154383-155084	DNA breaking-rejoining enzyme, phage integrase family (pfam00589)
	178	155855-155319	Hypothetical chloroplast-encoded protein of *Prasiola crispa* and *Mankyua chejuensis*

Supplementary Table S2. Non-canonical genes identified as freestanding ORFs in the ulvophycean chloroplast genomes examined in this study. [a]Reported here are the freestanding ORFs larger than 100 codons that revealed similarity (*E*-value threshold of 1e-06) with proteins of known functions and/or recognized protein domains in BlastP searches. Each ORF is identified by the number of amino acid residues in the encoded protein. [b]Genomic coordinates of the ORFs in the GenBank accessions.

Number	Sequence	Strand	Genomic position (3' end)
Ignatius tetrasporus			
1	GATGTATCAACGTCAAATCACG	+	183754
2	GATCTAGGGCTTGCTTTGCATTC	-	186657
3	TTAAAGCGATTGAGCGAAGCAG	+	188149
4	TAGAGGTATCCGATTTGCTCAG	-	192460
5	GTGACTTTAACCATGCGATGAG	-	451
6	AAGGAGTGTTGCGACTACAAAC	+	237146
7	TGCAAATAGTACTCAAAGCGTC	-	235837
8	TAAAGCTGATGGAAGACGTTTC	+	231801
Pseudoneochloris marina			
1	CAACCTACACAATCTTCCGTAC	-	103470
2	CTCGAAAGCTTGAACGTTGAAC	+	95139
3	GTAGCACCAGCTATTCAACCTC	+	128692
4	GAGTCGCACGAAAACAAGAGTC	-	419
5	AACAGGTTACTGCCTAAGGACG	+	129287
5	AACAGGTTACTGCCTAAGGACG	-	102782
6	GATGCTTTCAGCGATTATCCAC	+	95917
6	GATGCTTTCAGCGATTATCCAC	-	134412

Supplementary Table S3. Sequences of the PCR primers used to investigate flip-flop recombination in the *Ignatius* and *Pseudoneochloris* chloroplast genomes.

**Taxon**	**Collection**	**GenBank accession(s)**
**Streptophytes**		
Mesostigma viride	NIES 296	NC_002186
Chlorokybus atmophyticus	SAG 48.80	NC_008822
**Prasinophytes**		
Prasinococcus sp. CCMP 1194	CCMP 1194	KJ746597
Prasinophyceae sp. MBIC 10622	NBRC 102842	KJ746602
Prasinoderma coloniale	CCMP 1220	NC_024817
Pyramimonas parkeae	CCMP 726	FJ493499
Monomastix sp.OKE-1	PC O'kelly	NC_012101
Ostreococcus tauri	OTTH0595	NC_008289
Micromonas commoda	RCC 299	NC_012575
Pycnococcus provasolii	CCMP 1203	NC_012097
Nephroselmis olivacea	NIES 484	NC_000927
Nephroselmis astigmatica	NIES 252	NC_024829
Picocystis salinarum	CCMP 1897	NC_024828
Prasinophyceae sp. CCMP 1205	CCMP 1205	KJ746601
**Chlorodendrophyceae**		
Scherfellia dubia	SAG 17.86	NC_029807
Tetraselmis sp. CCMP 881	CCMP 881	KU167097
**Pedinophyceae**		
Marsupiomonas sp. NIES 1824	NIES 1824	KM462870
Pedinomonas tuberculata	SAG 42.84	NC_025530
Pedinomonas minor	UTEX LB 1350	NC_016733
**Chlorellales**		
Parachlorella kessleri	SAG 211-11g	NC_012978
Dicloster acuatus	SAG 41.98	NC_025546
Marvania geminata	SAG 12.88	NC_025549
Pseudochloris wilhelmii	SAG 1.80	NC_025547
Chlorella vulgaris		NC_001865
Chlorella variabilis	NC 64A	NC_015359
**Core Trebouxiophyceae**		
Oocystis solitaria	SAG 83.80	FJ968739
Planctonema lauterbornii	SAG 68.94	NC_025541
Koliella corcontica	SAG 24.84	NC_025536
Geminella terricola	SAG 20.91	NC_025542
Geminella minor	SAG 22.88	NC_025544
Gloeotilopsis sterilis	UTEX 1704	NC_025538
Pleurastrosarcina brevispinosa	UTEX 1176	KM462875
Parietochloris pseudoalveolaris	UTEX 975	NC_025532
Neocystis brevis	CAUP D802	NC_025535
Stichococcus bacillaris	UTEX 176	NC_025527
Prasiolopsis sp. SAG 84.81	SAG 84.81	KM462862
Chlorella mirabilis	SAG 38.88	NC_025528
Koliella longiseta	UTEX 339	NC_025531
Pabia signiensis	SAG 7.90	NC_025529
Leptospira terrestris	UTEX 333	NC_009681
Xylochloris irregularis	CAUP H7801	NC_025534
Trebouxia aggregata	SAG 219-1D	EU123962-EU124002
Myrmecia israelensis	UTEX 1181	NC_025525
Characium perforatum	SAG 28.85	NC_025543
Microthamnion kuetzingianum	UTEX 318	NC_025537
Lobosphaera incisa	SAG 2007	NC_025533
Watanabea reniformis	SAG 211-9b	NC_025526
Dictyochloropsis reticulata	SAG 2150	NC_025524
Choricystis minor	SAG 17.98	NC_025539
Botryococcus braunii	SAG 807-1	NC_025545
Elliptochloris bilobata	CAUP H7103	NC_025548
Trebouxiophyceae sp. MX-AZ01	MX-AZ01	NC_018569

Coccomyxa subellipsoidea	NIES 2166	NC_015084
Paradoxia multiseta	SAG 18.84	NC_025540
**Bryopsidales**		
Tydemania expeditionis	FL1151	NC_026796
Bryopsis plumosa	West4718	NC_026795
Bryopsis hypnoides		NC_013359
**Ignatiales**		
Pseudocharacium americanum	UTEX 2112	KY407658
Ignatius tetrasporus	UTEX 2012	KY407659
**Oltmannsiellopsidales**		
Dangemannia microcystis	SAG 2022	KY407660
Oltmannsiellopsis viridis	NIES 360	NC_008099
**Ulvales**		
Pseudoneochloris marina	UTEX 1445	KY407657
Ulva fasciata		NC_029040
Ulva sp. UNA	UNA00071828	KP720616
**Ulotrichales**		
Chamaetrichon capsulatum	UTEX 1918	KY407661
Pseudendoclonium akinetum	UTEX 1912	NC_008114
Trichosarcina mucosa	SAG 4.90	KY407656
Gloeotilopsis planctonica	SAG 29.93	KX306824
Gloeotilopsis sarcinoidea	UTEX 1710	KX306821
**Chlorophyceae**		
Floydiella terrestris	UTEX 1709	NC_014346
Oedogonium cardiacum	SAG 575-1b	NC_011031
Stigeoclonium helveticum	UTEX 441	NC_008372
Schizomeris leibleinii	UTEX LB 1228	NC_015645
Treubaria triappendiculata	SAG 38.83	NC_028578
Golenkinia longispicula	SAG 73.80	KT625092 - KT625150
Jenufa minuta	CAUP H8102	NC_028582
Jenufa perforata	CAUP H 8101	NC_028581
Bracteacoccus giganteus	UTEX 1251	NC_028586
Mychonastes jurisii	SAG 37.98	NC_028579
Acutodesmus obliquus	UTEX 393	NC_008101
Hafniomonas laevis	NIES 257	NC_028583
Carteria cerasiformis	NIES 425	NC_028585
Carteria crucifera	UTEX 432	KT624870 - KT624932
Carteria sp. SAG 8-5	SAG 8-5	KT625419
Chloromonas typhlos	UTEX LB 1969	KT624630 - KT624716
Chloromonas radiata	UTEX 966	KT625008 - KT625084
Oogamochlamys gigantea	SAG 44.91	NC_028580
Lobochlamys segnis	SAG 9.83	KT624806 - KT624869
Lobochlamys culleus	SAG 19.72	KT625151 - KT625204
Chlamydomonas asymmetrica	SAG 70.72	KT624933 - KT625007
Volvox carteri f. nagariensis	UTEX 2908	GU084820
Chlamydomonas reinhardtii		NC_005353
Phacotus lenticularis	SAG 61-1	NC_028587
Microglena monadina	SAG 31.72	KT624717 - KT624805
Chlamydomonas moewusii	UTEX 97	EF587443-EF587503
Characiochloris acuminata	SAG 31.95	NC_028584
Dunaliella salina	CCAP 19/18	NC_016732
Chlamydomonas applanata	SAG 11-9	KT625417
Stephanosphaera pluvialis	SAG 78-1a	KT625299 - KT625409
Chloromonas perforata	SAG 11-43	KT625416
Haematococcus lacustris	SAG 34-1b	KT625205 - KT625298
Chlorogonium capillatum	UTEX 11	KT625085 - KT625091

Supplementary Table S4. GenBank accession codes of the chloroplast genome sequences used to infer phylogenomic trees.

Supplementary Figure S1. Gene map of the *Ignatius tetrasporus* chloroplast genome. Filled boxes represent genes, with colors denoting gene categories as indicated in the legend. Genes on the outside are transcribed counterclockwise; those on the inside are transcribed clockwise.

Supplementary Figure S2. Gene map of the *Pseudocharacium americanum* chloroplast genome. Filled boxes represent genes, with colors denoting gene categories as indicated in the legend. Genes on the outside are transcribed counterclockwise; those on the inside are transcribed clockwise.

Supplementary Figure S3. Gene map of the *Dangemannia microcystis* chloroplast genome. Filled boxes represent genes, with colors denoting gene categories as indicated in the legend. Genes on the outside are transcribed counterclockwise; those on the inside are transcribed clockwise.

Supplementary Figure S4. Gene map of the *Pseudoneochloris marina* chloroplast genome. Filled boxes represent genes, with colors denoting gene categories as indicated in the legend. Genes on the outside are transcribed counterclockwise; those on the inside are transcribed clockwise.

Supplementary Figure S5. Gene map of the *Chamaetrichon capsulatum* chloroplast genome. Filled boxes represent genes, with colors denoting gene categories as indicated in the legend. Genes on the outside map are transcribed counterclockwise; those on the inside are transcribed clockwise.

Supplementary Figure S6. Gene map of the *Trichosarcina mucosa* chloroplast genome. Filled boxes represent genes, with colors denoting gene categories as indicated in the legend. Genes on the outside are transcribed counterclockwise; those on the inside are transcribed clockwise.

Supplementary Figure S7. Gene locations of the rapidly-evolving sites that were removed from the PCG-AA (1,303 sites) and PCG12RNA (4,492 sites) data sets by filtration with TIGER.

Supplementary Figure S8. Phylogenomic trees inferred from the PCG12RNA data set using RAxML. Bootstrap support values are reported on the nodes. A black dot indicates that the corresponding branch received a value of 100%. The scale bar denotes the estimated number of substitutions per site.

Supplementary Figure S9. Phylogenomic trees inferred from the PCG12RNA data set using PhyloBayes. Posterior probability values are reported on the nodes. A black dot indicates that the corresponding branch received a value of 1.0. The scale bar denotes the estimated number of substitutions per site.

Supplementary Figure S10. Phylogenomic trees inferred from the PCG-AA data set using RAxML. Bootstrap support values are reported on the nodes. A black dot indicates that the corresponding branch received a value of 100%. The scale bar denotes the estimated number of substitutions per site.

Supplementary Figure S11. Phylogenomic trees inferred from the PCG-AA data set using PhyloBayes. Posterior probability values are reported on the nodes. A black dot

indicates that the corresponding branch received a value of 1.0. The scale bar denotes the estimated number of substitutions per site.

Supplementary Figure S12. Repertoires of canonical genes in the ulvophycean chloroplast genomes compared in this study. Only the genes missing in one or more genomes are indicated. The presence of genes and pseudogenes is denoted by blue and light blue boxes, respectively. Protein-coding genes that were lost only once are mapped on the cladogram. A total of 95 genes are shared by all compared genomes: *accD, atpA, B, E, F, H, I, ccsA, cemA, chlI, clpP, ftsH, infA, petA, B, D, G, L, psaA, B, C, I, J, M, psbA, B, C, D, E, F, H, I, J, K, L, M, N, T, Z, rbcL, rpl2, 5, 14, 16, 19, 20, 23, 32, 36, rpoA, C2, rps2, 3, 4, 7, 8, 9, 11, 12, 14, 18, 19, rrf, rrl, rrs, tufA, ycf1, 3, 4, 12, trnA*(ugc), *C*(gca), *D*(guc), *E*(uuc), *F*(gaa), *G*(gcc), *G*(ucc), *H*(gug), *I*(gau), *K*(uuu), *L*(uaa), *L*(uag), *Me*(cau), *Mf*(cau), *N*(guu), *P*(ugg), *Q*(uug), *R*(acg), *R*(ucu), *S*(gcu), *S*(uga), *T*(ugu), *V*(uac), *W*(cca), *Y*(gua).

References

1 Lemieux, C., Otis, C. & Turmel, M. Ancestral chloroplast genome in *Mesostigma viride* reveals an early branch of green plant evolution. *Nature* **403**, 649-652, doi:10.1038/35001059 (2000).

2 Turmel, M., Otis, C. & Lemieux, C. Mitochondrion-to-chloroplast DNA transfers and intragenomic proliferation of chloroplast group II introns in *Gloeotilopsis* green algae (Ulotrichales, Ulvophyceae). *Genome Biol Evol* **8**, 2789-2805, doi:10.1093/gbe/evw190 (2016).

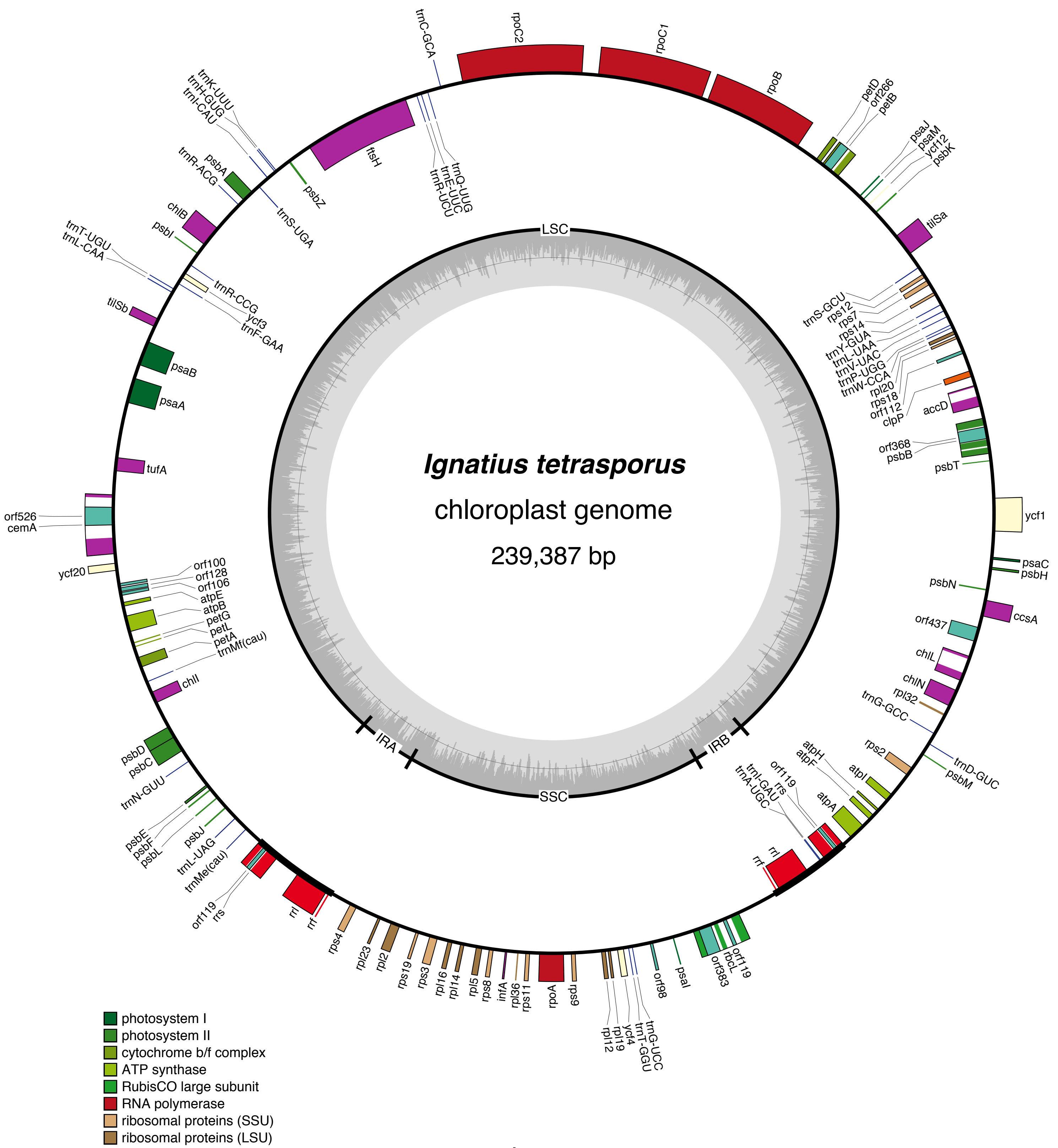
Ignatius tetrasporus
chloroplast genome
239,387 bp
LSC
IRA
IRB
SSC
rpoC2
rpoC1
rpoB
trnC-GCA
ftsH
trnQ-UUG
trnE-UUC
trnR-UCU
psbZ
trnS-UGA
trnK-UUU
trnH-GUG
trnI-CAU
psbA
trnR-ACG
chlB
psbI
trnT-UGU
trnL-CAA
trnR-CCG
ycf3
trnF-GAA
tilSb
psaB
psaA
tufA
orf526
cemA
ycf20
orf100
orf128
orf106
atpE
atpB
petG
petL
petA
trnMf(cau)
chlI
psbD
psbC
trnN-GUU
psbE
psbF
psbL
psbJ
trnL-UAG
trnMe(cau)
orf119
rrs
rrl
rrf
rps4
rpl23
rpl2
rps19
rps3
rpl16
rpl14
rpl5
rps8
infA
rpl36
rps11
rpoA
rps9
rpl12
rpl19
ycf4
trnT-GGU
trnG-UCC
orf98
psaI
orf383
rbcL
orf119
petD
orf266
petB
psaJ
psaM
ycf12
psbK
tilSa
trnS-GCU
rps12
rps7
rps14
trnY-GUA
trnL-UAA
trnV-UAC
trnP-UGG
trnW-CCA
rpl20
rps18
orf112
clpP
accD
orf368
psbB
psbT
ycf1
psaC
psbH
psbN
ccsA
orf437
chlL
chlN
rpl32
trnG-GCC
trnD-GUC
psbM
rps2
atpH
atpF
atpI
atpA
orf119
rrs
trnI-GAU
trnA-UGC
rrl
rrf
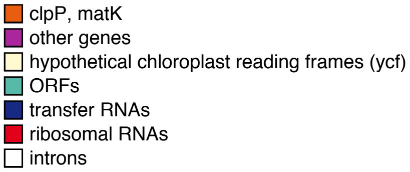
photosystem I
photosystem II
cytochrome b/f complex
ATP synthase
RubisCO large subunit
RNA polymerase
ribosomal proteins (SSU)
ribosomal proteins (LSU)
clpP, matK
other genes
hypothetical chloroplast reading frames (ycf)
ORFs
transfer RNAs
ribosomal RNAs
introns

Figure S1

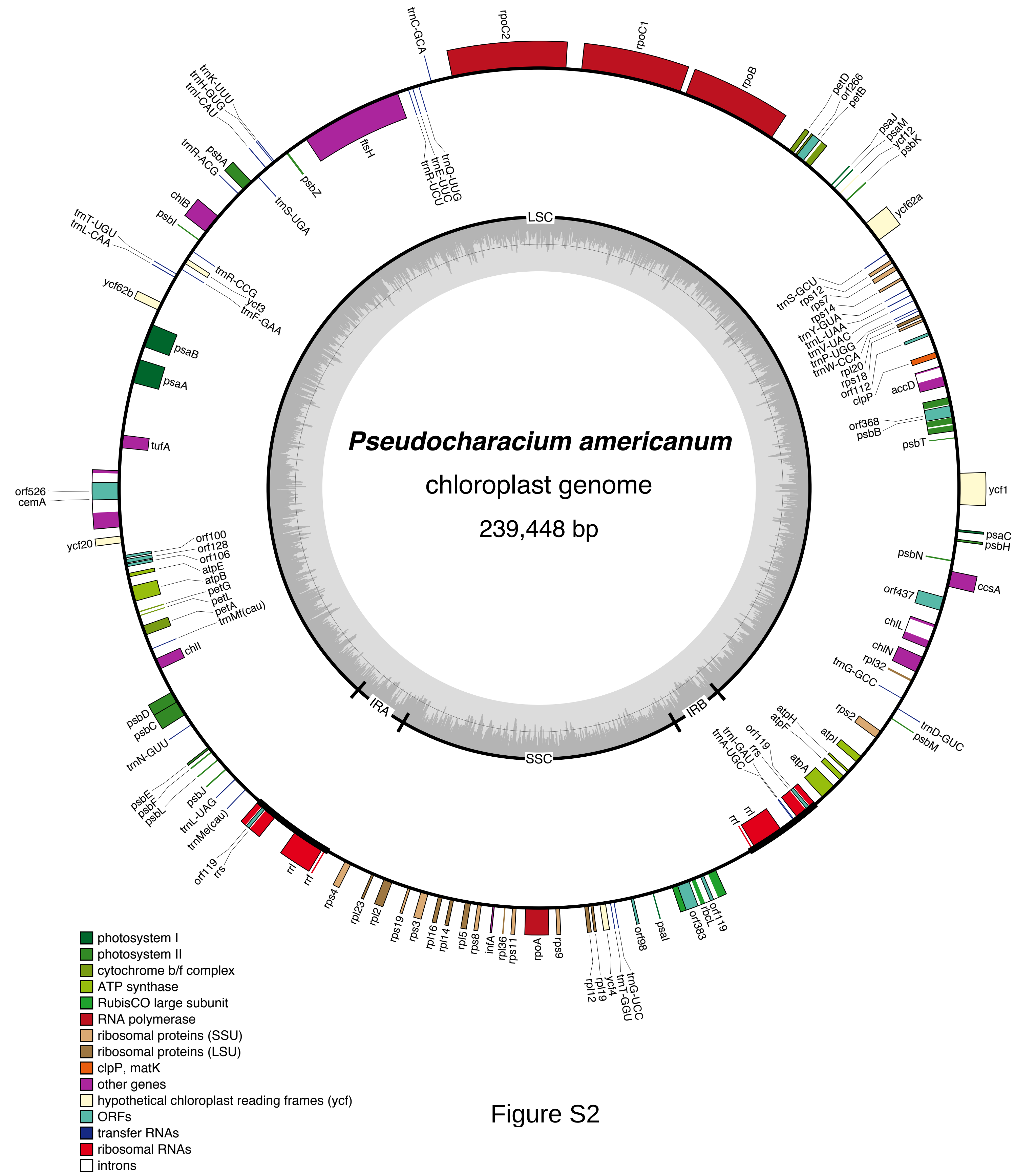

Pseudocharacium americanum
chloroplast genome
239,448 bp
LSC
IRA
IRB
SSC
rpoC2
rpoC1
rpoB
ftsH
psbA
chlB
psaB
psaA
tufA
cemA
ycf1
ccsA
rbcL
rpoA
photosystem I
photosystem II
cytochrome b/f complex
ATP synthase
RubisCO large subunit
RNA polymerase
ribosomal proteins (SSU)
ribosomal proteins (LSU)
clpP, matK
other genes
hypothetical chloroplast reading frames (ycf)
ORFs
transfer RNAs
ribosomal RNAs
introns

Figure S2

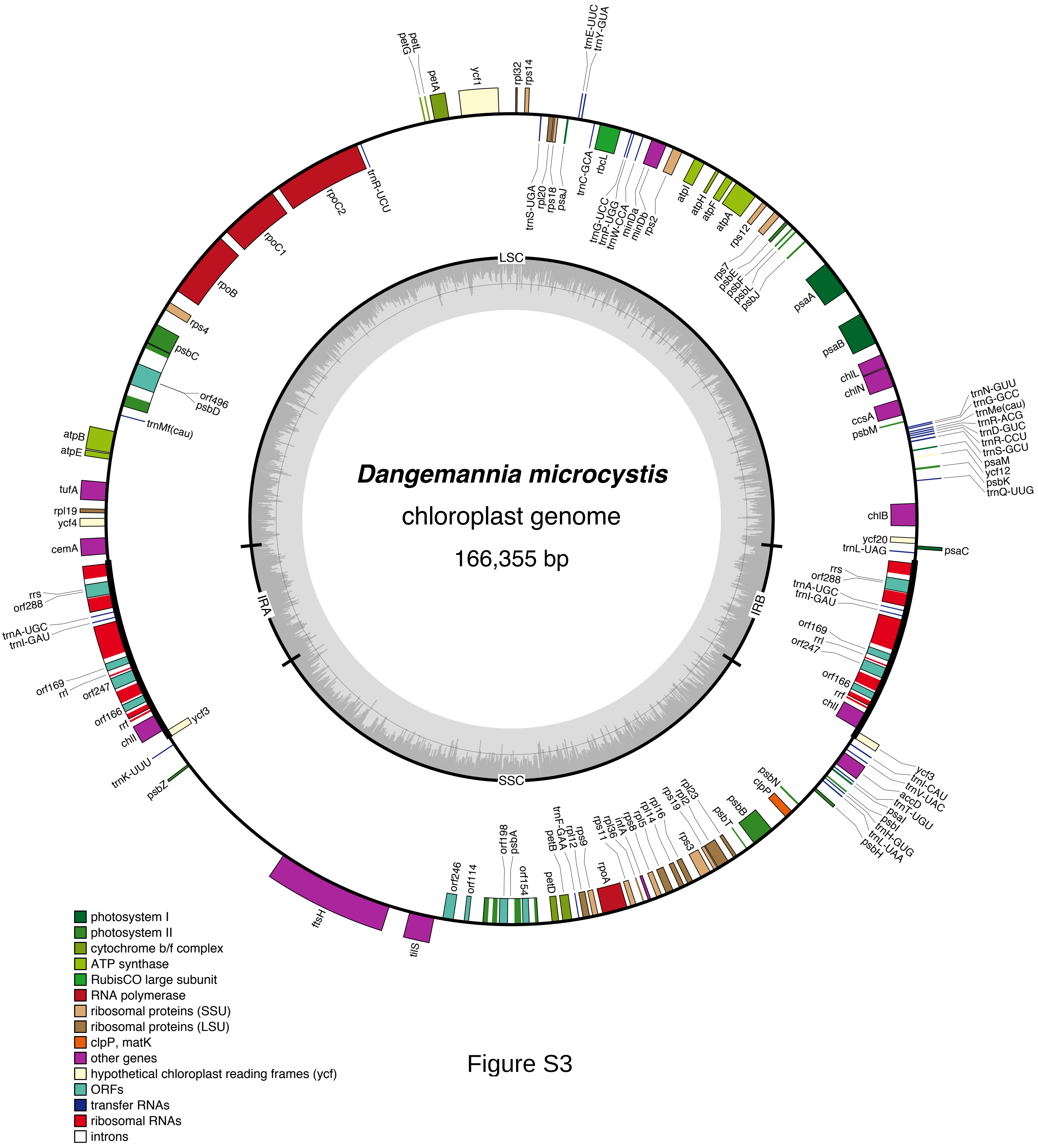
Dangemannia microcystis
chloroplast genome
166,355 bp
LSC
IRB
SSC
IRA
trnE-UUC
trnY-GUA
trnS-UGA
rpl20
rps18
psaJ
trnC-GCA
rbcL
trnG-UCC
trnP-UGG
trnW-CCA
minDa
minDb
rps2
atpI
atpH
atpF
atpA
rps12
rps7
psbE
psbF
psbL
psbJ
psaA
psaB
chlL
chlN
ccsA
psbM
trnN-GUU
trnG-GCC
trnMe(cau)
trnR-ACG
trnD-GUC
trnR-CCU
trnS-GCU
psaM
ycf12
psbK
trnQ-UUG
chlB
ycf20
trnL-UAG
psaC
rrs
orf288
trnA-UGC
trnI-GAU
orf169
rrl
orf247
orf166
rrf
chlI
ycf3
trnI-CAU
trnV-UAC
accD
trnT-UGU
psaI
psbI
trnH-GUG
trnL-UAA
psbH
psbN
clpP
psbB
psbT
rpl23
rpl2
rps19
rps3
rpl16
rpl14
rpl5
rps8
infA
rpl36
rps11
rpoA
rps9
rpl12
trnF-GAA
petB
petD
orf154
orf198
psbA
orf114
orf246
tilS
ftsH
psbZ
trnK-UUU
ycf3
chlI
rrf
orf166
orf247
rrl
orf169
trnA-UGC
trnI-GAU
rrs
orf288
cemA
ycf4
rpl19
tufA
atpE
atpB
trnMf(cau)
orf496
psbD
psbC
rps4
rpoB
rpoC1
rpoC2
trnR-UCU
petL
petG
petA
ycf1
rpl32
rps14
photosystem I
photosystem II
cytochrome b/f complex
ATP synthase
RubisCO large subunit
RNA polymerase
ribosomal proteins (SSU)
ribosomal proteins (LSU)
clpP, matK
other genes
hypothetical chloroplast reading frames (ycf)
ORFs
transfer RNAs
ribosomal RNAs
introns

Figure S3

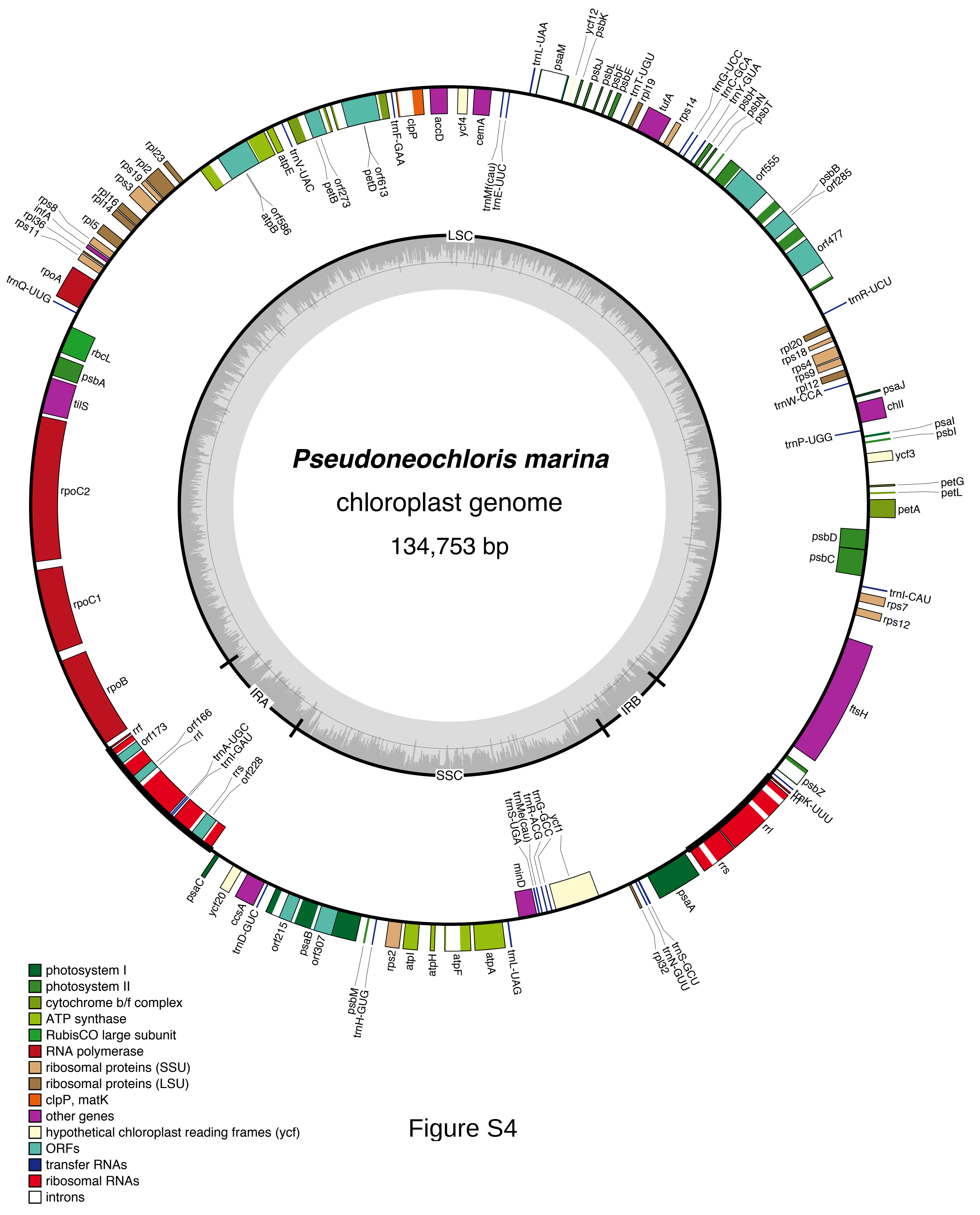

Pseudoneochloris marina
chloroplast genome
134,753 bp
LSC
IRA
IRB
SSC
trnL-UAA
psaM
ycf12
psbK
psbJ
psbL
psbF
psbE
trnT-UGU
rpl19
tufA
rps14
trnG-UCC
trnC-GCA
trnY-GUA
psbH
psbN
psbT
orf555
psbB
orf285
orf477
trnR-UCU
rpl20
rps18
rps4
rps9
rpl12
trnW-CCA
psaJ
chlI
trnP-UGG
psaI
psbI
ycf3
petG
petL
petA
psbD
psbC
trnI-CAU
rps7
rps12
ftsH
psbZ
trnK-UUU
rrf
rrl
rrs
psaA
trnS-GCU
trnN-GUU
rpl32
trnL-UAG
atpA
atpF
atpH
atpI
rps2
trnH-GUG
psbM
orf307
psaB
orf215
trnD-GUC
ccsA
ycf20
psaC
minD
trnS-UGA
trnMe(cau)
trnR-ACG
trnG-GCC
ycf1
orf228
rrs
trnI-GAU
trnA-UGC
rrl
orf166
orf173
rrf
rpoB
rpoC1
rpoC2
tilS
psbA
rbcL
trnQ-UUG
rpoA
rps11
rpl36
infA
rps8
rpl5
rpl14
rpl16
rps3
rps19
rpl2
rpl23
atpB
orf586
atpE
trnV-UAC
petB
orf273
petD
orf613
trnF-GAA
clpP
accD
ycf4
cemA
trnMf(cau)
trnE-UUC
photosystem I
photosystem II
cytochrome b/f complex
ATP synthase
RubisCO large subunit
RNA polymerase
ribosomal proteins (SSU)
ribosomal proteins (LSU)
clpP, matK
other genes
hypothetical chloroplast reading frames (ycf)
ORFs
transfer RNAs
ribosomal RNAs
introns

Figure S4

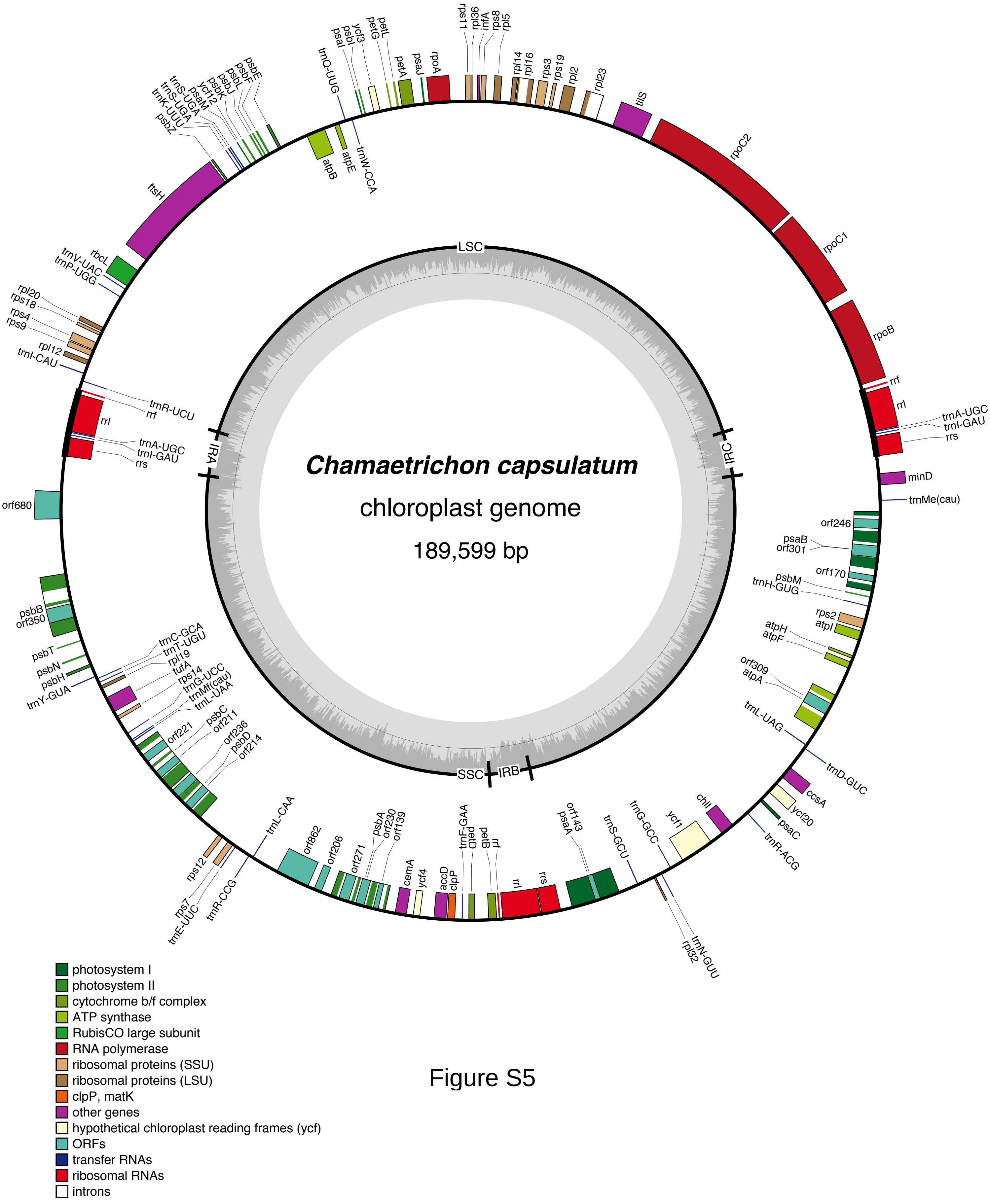
Chamaetrichon capsulatum
chloroplast genome
189,599 bp
LSC
IRA
IRB
IRC
SSC
rpoC2
rpoC1
rpoB
tilS
ftsH
rbcL
orf680
minD
orf862
rrl
rrs
photosystem I
photosystem II
cytochrome b/f complex
ATP synthase
RubisCO large subunit
RNA polymerase
ribosomal proteins (SSU)
ribosomal proteins (LSU)
clpP, matK
other genes
hypothetical chloroplast reading frames (ycf)
ORFs
transfer RNAs
ribosomal RNAs
introns

Figure S5

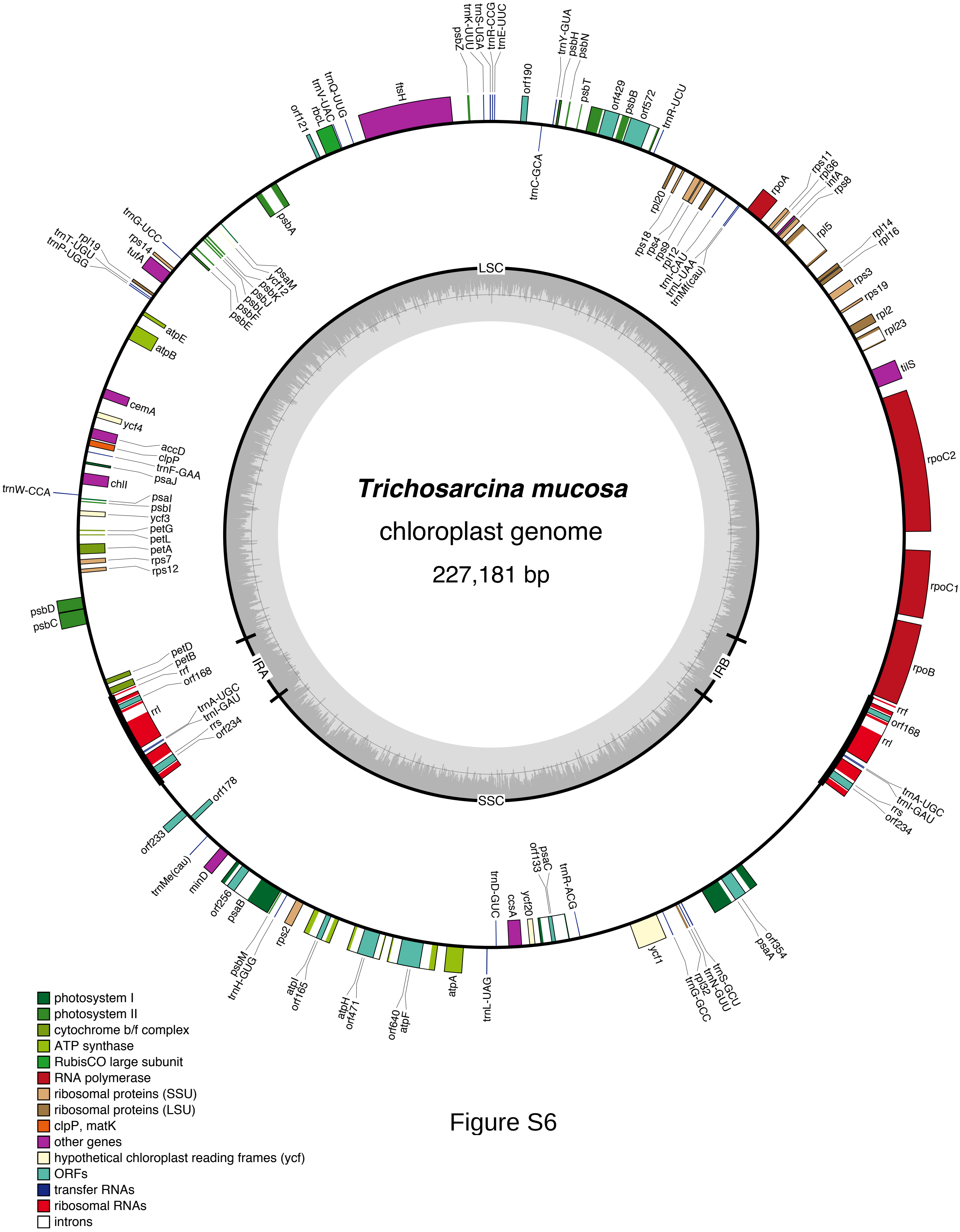
Trichosarcina mucosa
chloroplast genome
227,181 bp
LSC
SSC
IRA
IRB
psbZ
trnK-UUU
trnS-UGA
trnR-CCG
trnE-UUC
orf190
trnY-GUA
psbH
psbN
psbT
orf429
psbB
orf572
trnR-UCU
trnC-GCA
ftsH
trnQ-UUG
trnV-UAC
rbcL
orf121
psbA
rpl20
rps18
rps4
rps9
rpl12
trnI-CAU
trnL-UAA
trnMf(cau)
rpoA
rps11
rpl36
infA
rps8
rpl5
rpl14
rpl16
rps3
rps19
rpl2
rpl23
tilS
rpoC2
rpoC1
rpoB
rrf
orf168
rrl
trnA-UGC
trnI-GAU
rrs
orf234
trnG-UCC
rps14
tufA
rpl19
trnT-UGU
trnP-UGG
psaM
ycf12
psbK
psbJ
psbL
psbF
psbE
atpE
atpB
cemA
ycf4
accD
clpP
trnF-GAA
psaJ
chlI
trnW-CCA
psaI
psbI
ycf3
petG
petL
petA
rps7
rps12
psbD
psbC
petD
petB
orf178
orf233
trnMe(cau)
minD
orf256
psaB
psbM
trnH-GUG
rps2
atpI
orf165
atpH
orf471
orf640
atpF
atpA
trnL-UAG
trnD-GUC
ccsA
ycf20
psaC
orf133
trnR-ACG
ycf1
trnG-GCC
rpl32
trnN-GUU
trnS-GCU
psaA
orf354
photosystem I
photosystem II
cytochrome b/f complex
ATP synthase
RubisCO large subunit
RNA polymerase
ribosomal proteins (SSU)
ribosomal proteins (LSU)
clpP, matK
other genes
hypothetical chloroplast reading frames (ycf)
ORFs
transfer RNAs
ribosomal RNAs
introns

Figure S6

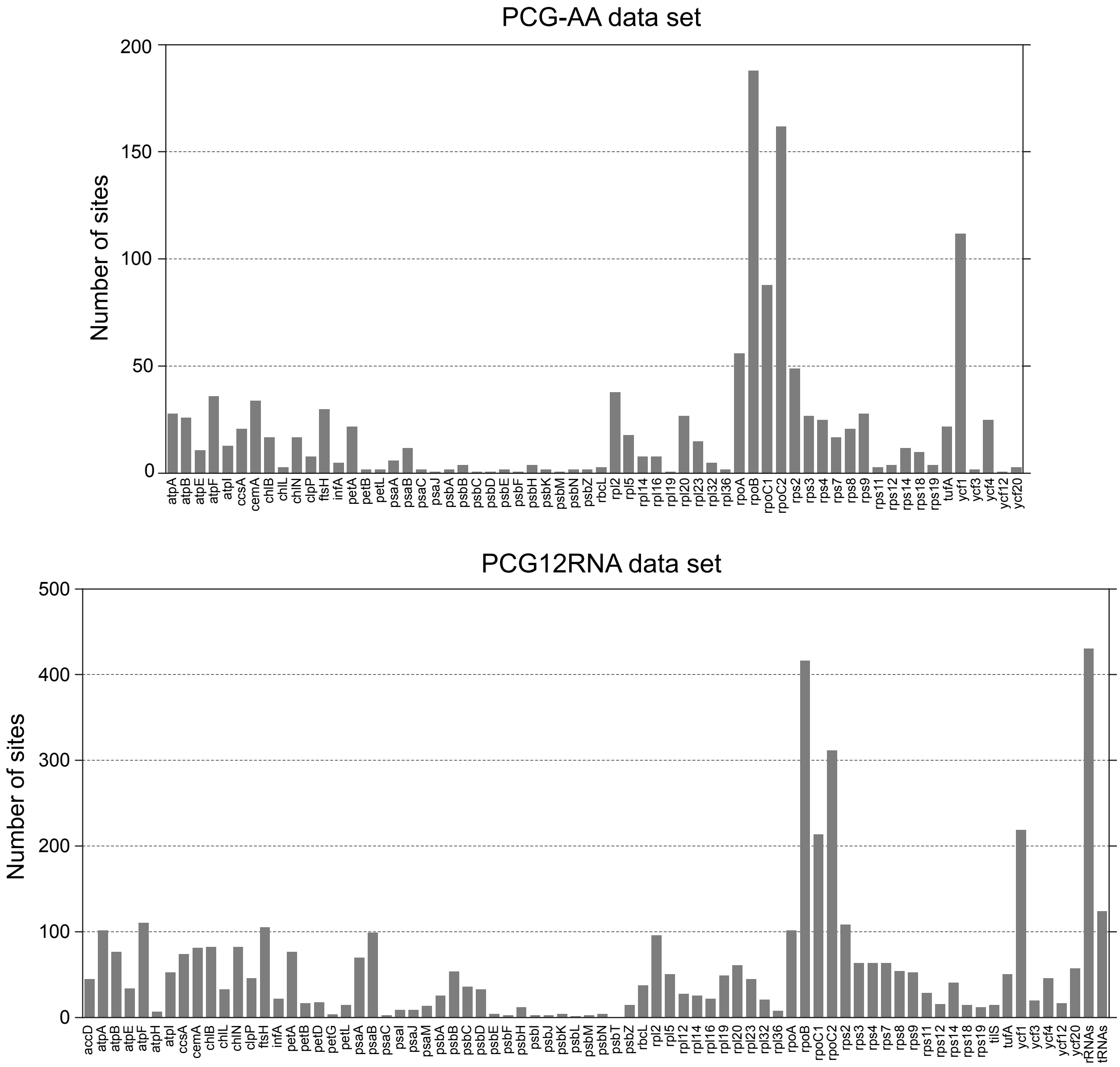
PCG-AA data set
Number of sites
200
150
100
50
0
atpA atpB atpE atpF atpI ccsA cemA chlB chlL chlN clpP ftsH infA petA petB petL psaA psaB psaC psaJ psbA psbB psbC psbD psbE psbF psbH psbK psbM psbN psbZ rbcL rpl2 rpl5 rpl14 rpl16 rpl19 rpl20 rpl23 rpl32 rpl36 rpoA rpoB rpoC1 rpoC2 rps2 rps3 rps4 rps7 rps8 rps9 rps11 rps12 rps14 rps18 rps19 tufA ycf1 ycf3 ycf4 ycf12 ycf20
PCG12RNA data set
Number of sites
500
400
300
200
100
0
accD atpA atpB atpE atpF atpH atpI ccsA cemA chlB chlL chlN clpP ftsH infA petA petB petD petG petL psaA psaB psaC psaI psaJ psaM psbA psbB psbC psbD psbE psbF psbH psbI psbJ psbK psbL psbM psbN psbT psbZ rbcL rpl2 rpl5 rpl12 rpl14 rpl16 rpl19 rpl20 rpl23 rpl32 rpl36 rpoA rpoB rpoC1 rpoC2 rps2 rps3 rps4 rps7 rps8 rps9 rps11 rps12 rps14 rps18 rps19 tilS tufA ycf1 ycf3 ycf4 ycf12 ycf20 rRNAs tRNAs

Figure S7

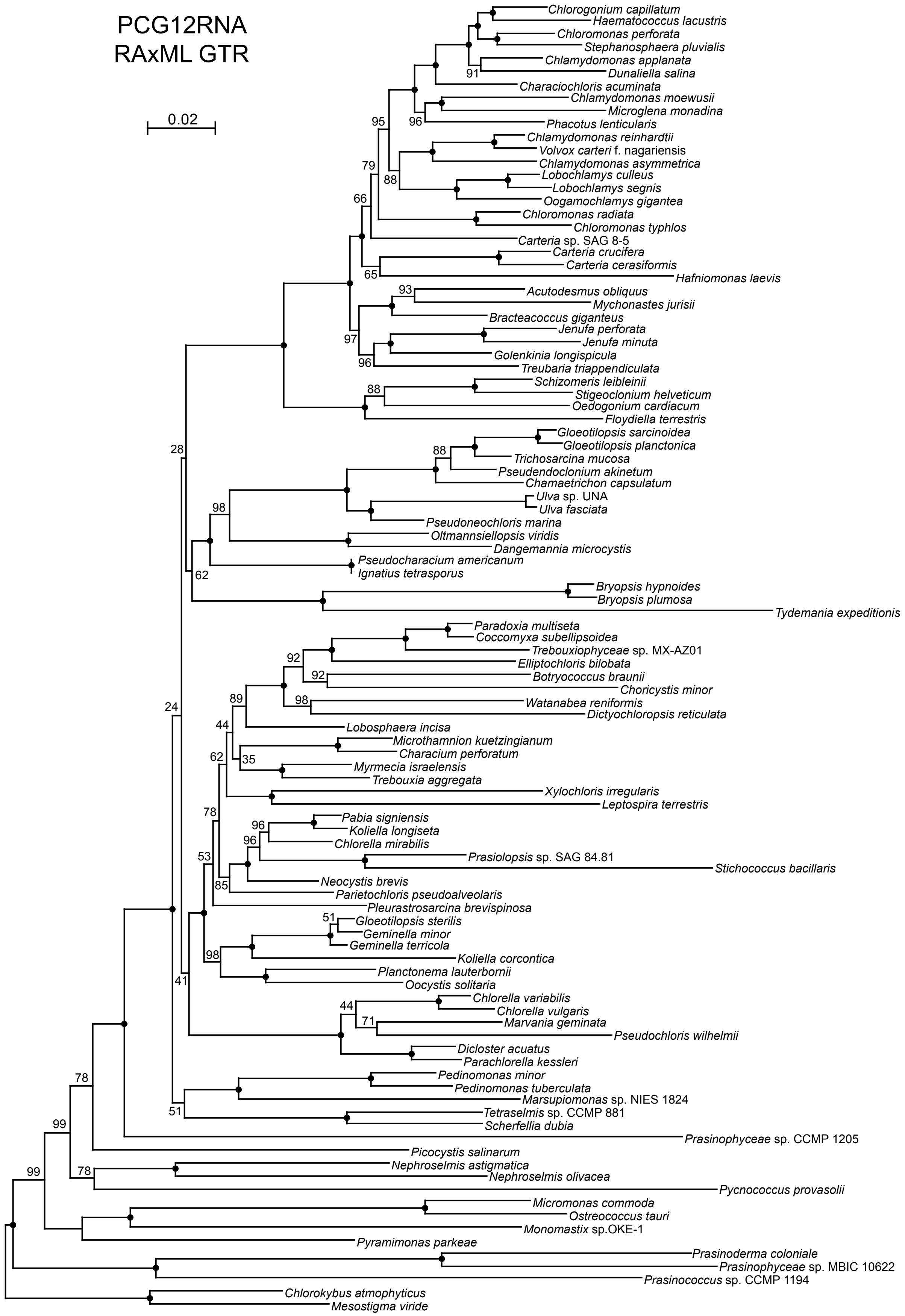
PCG12RNA
RAxML GTR
0.02
Chlorogonium capillatum
Haematococcus lacustris
Chloromonas perforata
Stephanosphaera pluvialis
Chlamydomonas applanata
Dunaliella salina
Characiochloris acuminata
Chlamydomonas moewusii
Microglena monadina
Phacotus lenticularis
Chlamydomonas reinhardtii
Volvox carteri f. nagariensis
Chlamydomonas asymmetrica
Lobochlamys culleus
Lobochlamys segnis
Oogamochlamys gigantea
Chloromonas radiata
Chloromonas typhlos
Carteria sp. SAG 8-5
Carteria crucifera
Carteria cerasiformis
Hafniomonas laevis
Acutodesmus obliquus
Mychonastes jurisii
Bracteacoccus giganteus
Jenufa perforata
Jenufa minuta
Golenkinia longispicula
Treubaria triappendiculata
Schizomeris leibleinii
Stigeoclonium helveticum
Oedogonium cardiacum
Floydiella terrestris
Gloeotilopsis sarcinoidea
Gloeotilopsis planctonica
Trichosarcina mucosa
Pseudendoclonium akinetum
Chamaetrichon capsulatum
Ulva sp. UNA
Ulva fasciata
Pseudoneochloris marina
Oltmannsiellopsis viridis
Dangemannia microcystis
Pseudocharacium americanum
Ignatius tetrasporus
Bryopsis hypnoides
Bryopsis plumosa
Tydemania expeditionis
Paradoxia multiseta
Coccomyxa subellipsoidea
Trebouxiophyceae sp. MX-AZ01
Elliptochloris bilobata
Botryococcus braunii
Choricystis minor
Watanabea reniformis
Dictyochloropsis reticulata
Lobosphaera incisa
Microthamnion kuetzingianum
Characium perforatum
Myrmecia israelensis
Trebouxia aggregata
Xylochloris irregularis
Leptosira terrestris
Pabia signiensis
Koliella longiseta
Chlorella mirabilis
Prasiolopsis sp. SAG 84.81
Stichococcus bacillaris
Neocystis brevis
Parietochloris pseudoalveolaris
Pleurastrosarcina brevispinosa
Gloeotilopsis sterilis
Geminella minor
Geminella terricola
Koliella corcontica
Planctonema lauterbornii
Oocystis solitaria
Chlorella variabilis
Chlorella vulgaris
Marvania geminata
Pseudochloris wilhelmii
Dicloster acuatus
Parachlorella kessleri
Pedinomonas minor
Pedinomonas tuberculata
Marsupiomonas sp. NIES 1824
Tetraselmis sp. CCMP 881
Scherffelia dubia
Prasinophyceae sp. CCMP 1205
Picocystis salinarum
Nephroselmis astigmatica
Nephroselmis olivacea
Pycnococcus provasolii
Micromonas commoda
Ostreococcus tauri
Monomastix sp.OKE-1
Pyramimonas parkeae
Prasinoderma coloniale
Prasinophyceae sp. MBIC 10622
Prasinococcus sp. CCMP 1194
Chlorokybus atmophyticus
Mesostigma viride
91
96
95
79
88
66
65
93
97
96
88
28
88
98
62
92
92
98
89
44
35
62
78
96
96
53
85
51
98
41
44
71
24
51
78
99
78
99

Figure S8

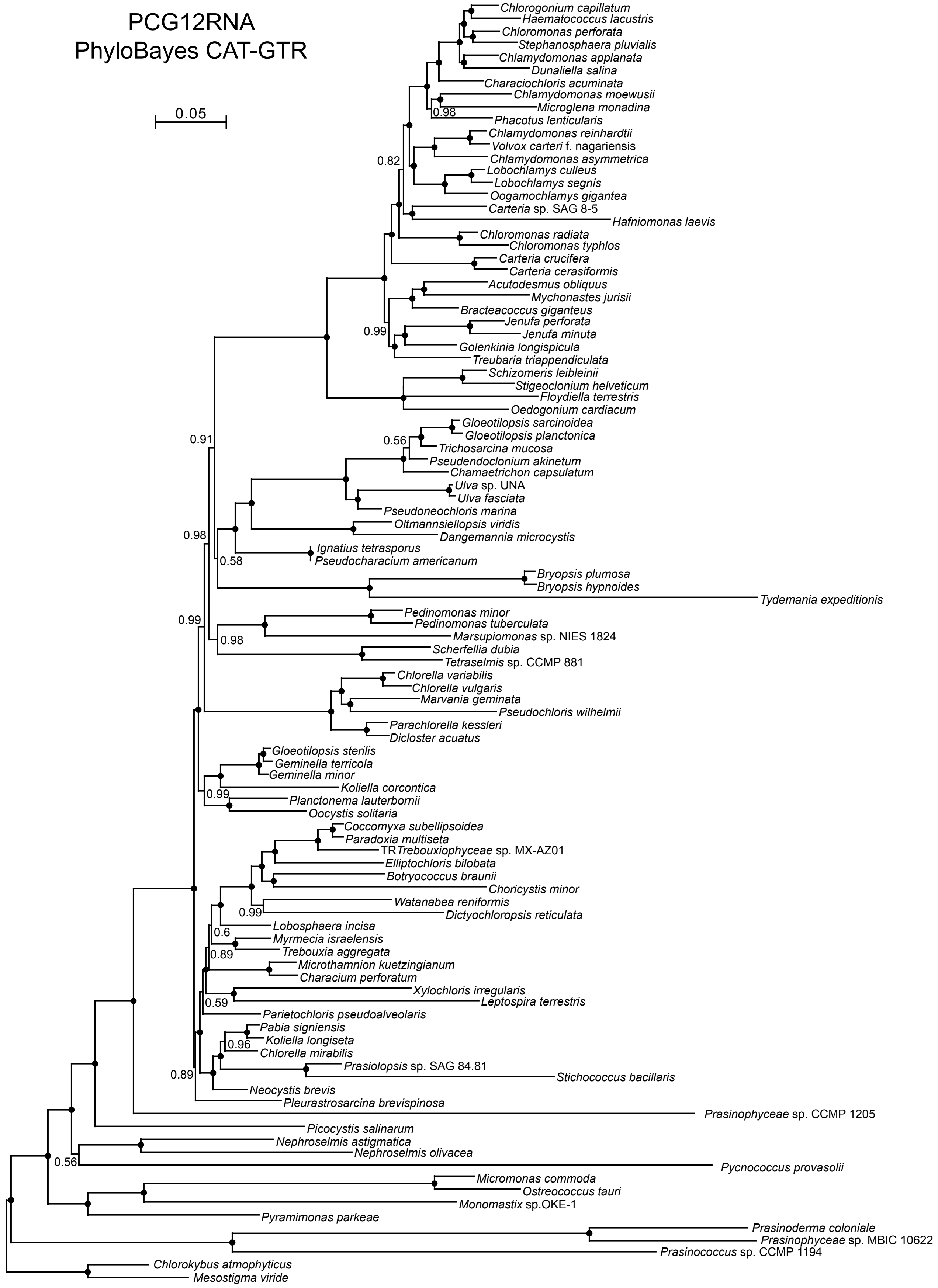
PCG12RNA
PhyloBayes CAT-GTR
0.05
Chlorogonium capillatum
Haematococcus lacustris
Chloromonas perforata
Stephanosphaera pluvialis
Chlamydomonas applanata
Dunaliella salina
Characiochloris acuminata
Chlamydomonas moewusii
0.98
Microglena monadina
Phacotus lenticularis
Chlamydomonas reinhardtii
Volvox carteri f. nagariensis
Chlamydomonas asymmetrica
0.82
Lobochlamys culleus
Lobochlamys segnis
Oogamochlamys gigantea
Carteria sp. SAG 8-5
Hafniomonas laevis
Chloromonas radiata
Chloromonas typhlos
Carteria crucifera
Carteria cerasiformis
Acutodesmus obliquus
Mychonastes jurisii
Bracteacoccus giganteus
0.99
Jenufa perforata
Jenufa minuta
Golenkinia longispicula
Treubaria triappendiculata
Schizomeris leibleinii
Stigeoclonium helveticum
Floydiella terrestris
Oedogonium cardiacum
0.91
Gloeotilopsis sarcinoidea
0.56
Gloeotilopsis planctonica
Trichosarcina mucosa
Pseudendoclonium akinetum
Chamaetrichon capsulatum
Ulva sp. UNA
Ulva fasciata
Pseudoneochloris marina
Oltmannsiellopsis viridis
Dangemannia microcystis
0.98
Ignatius tetrasporus
0.58
Pseudocharacium americanum
Bryopsis plumosa
Bryopsis hypnoides
Tydemania expeditionis
Pedinomonas minor
0.99
Pedinomonas tuberculata
Marsupiomonas sp. NIES 1824
0.98
Scherfellia dubia
Tetraselmis sp. CCMP 881
Chlorella variabilis
Chlorella vulgaris
Marvania geminata
Pseudochloris wilhelmii
Parachlorella kessleri
Dicloster acuatus
Gloeotilopsis sterilis
Geminella terricola
Geminella minor
Koliella corcontica
0.99
Planctonema lauterbornii
Oocystis solitaria
Coccomyxa subellipsoidea
Paradoxia multiseta
TRTrebouxiophyceae sp. MX-AZ01
Elliptochloris bilobata
Botryococcus braunii
Choricystis minor
Watanabea reniformis
0.99
Dictyochloropsis reticulata
Lobosphaera incisa
0.6
Myrmecia israelensis
0.89
Trebouxia aggregata
Microthamnion kuetzingianum
Characium perforatum
Xylochloris irregularis
0.59
Leptospira terrestris
Parietochloris pseudoalveolaris
Pabia signiensis
0.96
Koliella longiseta
Chlorella mirabilis
Prasiolopsis sp. SAG 84.81
0.89
Stichococcus bacillaris
Neocystis brevis
Pleurastrosarcina brevispinosa
Prasinophyceae sp. CCMP 1205
Picocystis salinarum
Nephroselmis astigmatica
Nephroselmis olivacea
0.56
Pycnococcus provasolii
Micromonas commoda
Ostreococcus tauri
Monomastix sp.OKE-1
Pyramimonas parkeae
Prasinoderma coloniale
Prasinophyceae sp. MBIC 10622
Prasinococcus sp. CCMP 1194
Chlorokybus atmophyticus
Mesostigma viride

Figure S9

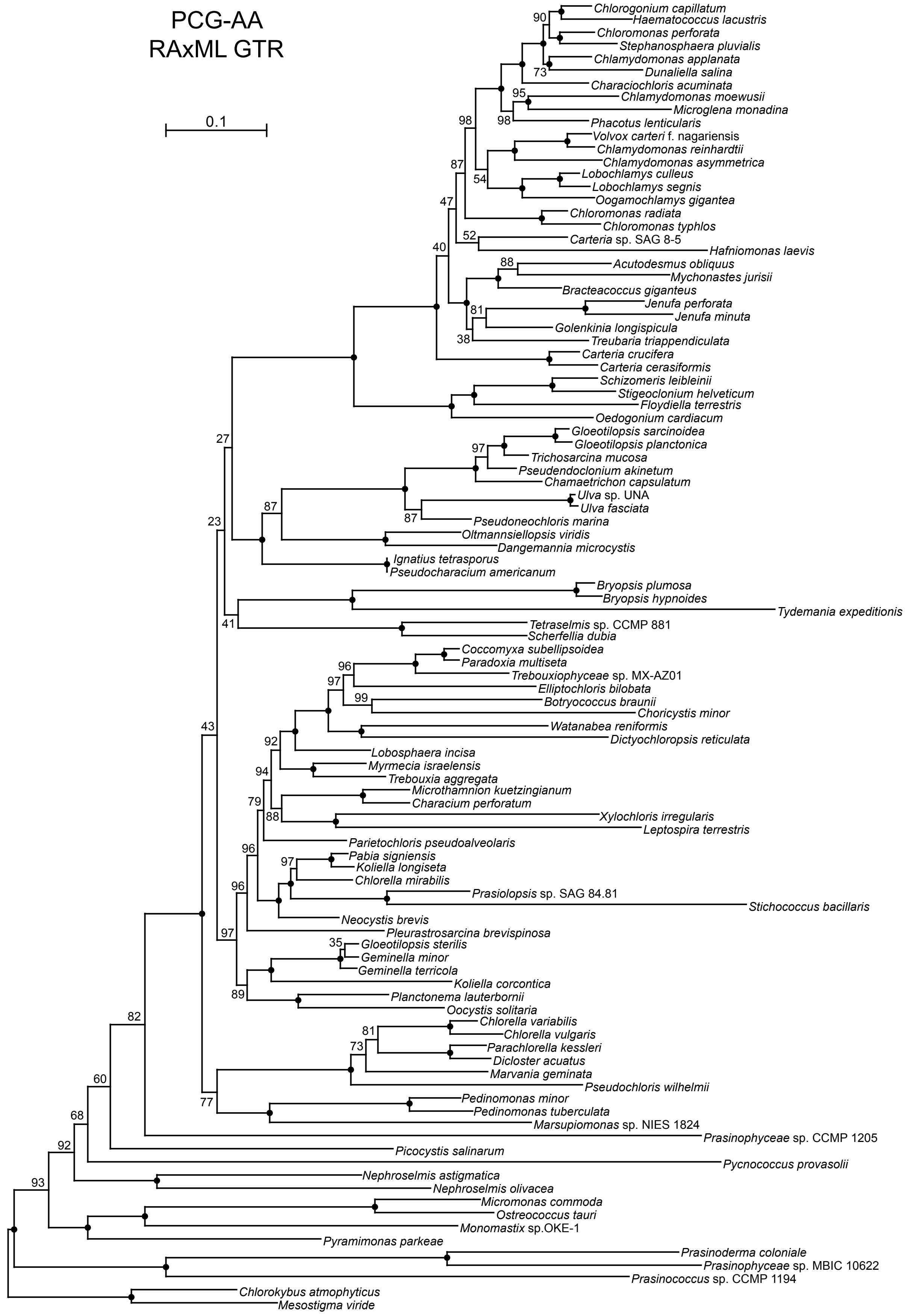
PCG-AA
RAxML GTR
0.1
Chlorogonium capillatum
Haematococcus lacustris
Chloromonas perforata
Stephanosphaera pluvialis
Chlamydomonas applanata
Dunaliella salina
Characiochloris acuminata
Chlamydomonas moewusii
Microglena monadina
Phacotus lenticularis
Volvox carteri f. nagariensis
Chlamydomonas reinhardtii
Chlamydomonas asymmetrica
Lobochlamys culleus
Lobochlamys segnis
Oogamochlamys gigantea
Chloromonas radiata
Chloromonas typhlos
Carteria sp. SAG 8-5
Hafniomonas laevis
Acutodesmus obliquus
Mychonastes jurisii
Bracteacoccus giganteus
Jenufa perforata
Jenufa minuta
Golenkinia longispicula
Treubaria triappendiculata
Carteria crucifera
Carteria cerasiformis
Schizomeris leibleinii
Stigeoclonium helveticum
Floydiella terrestris
Oedogonium cardiacum
Gloeotilopsis sarcinoidea
Gloeotilopsis planctonica
Trichosarcina mucosa
Pseudendoclonium akinetum
Chamaetrichon capsulatum
Ulva sp. UNA
Ulva fasciata
Pseudoneochloris marina
Oltmannsiellopsis viridis
Dangemannia microcystis
Ignatius tetrasporus
Pseudocharacium americanum
Bryopsis plumosa
Bryopsis hypnoides
Tydemania expeditionis
Tetraselmis sp. CCMP 881
Scherffelia dubia
Coccomyxa subellipsoidea
Paradoxia multiseta
Trebouxiophyceae sp. MX-AZ01
Elliptochloris bilobata
Botryococcus braunii
Choricystis minor
Watanabea reniformis
Dictyochloropsis reticulata
Lobosphaera incisa
Myrmecia israelensis
Trebouxia aggregata
Microthamnion kuetzingianum
Characium perforatum
Xylochloris irregularis
Leptospira terrestris
Parietochloris pseudoalveolaris
Pabia signiensis
Koliella longiseta
Chlorella mirabilis
Prasiolopsis sp. SAG 84.81
Stichococcus bacillaris
Neocystis brevis
Pleurastrosarcina brevispinosa
Gloeotilopsis sterilis
Geminella minor
Geminella terricola
Koliella corcontica
Planctonema lauterbornii
Oocystis solitaria
Chlorella variabilis
Chlorella vulgaris
Parachlorella kessleri
Dicloster acuatus
Marvania geminata
Pseudochloris wilhelmii
Pedinomonas minor
Pedinomonas tuberculata
Marsupiomonas sp. NIES 1824
Prasinophyceae sp. CCMP 1205
Picocystis salinarum
Pycnococcus provasolii
Nephroselmis astigmatica
Nephroselmis olivacea
Micromonas commoda
Ostreococcus tauri
Monomastix sp.OKE-1
Pyramimonas parkeae
Prasinoderma coloniale
Prasinophyceae sp. MBIC 10622
Prasinococcus sp. CCMP 1194
Chlorokybus atmophyticus
Mesostigma viride

Figure S10

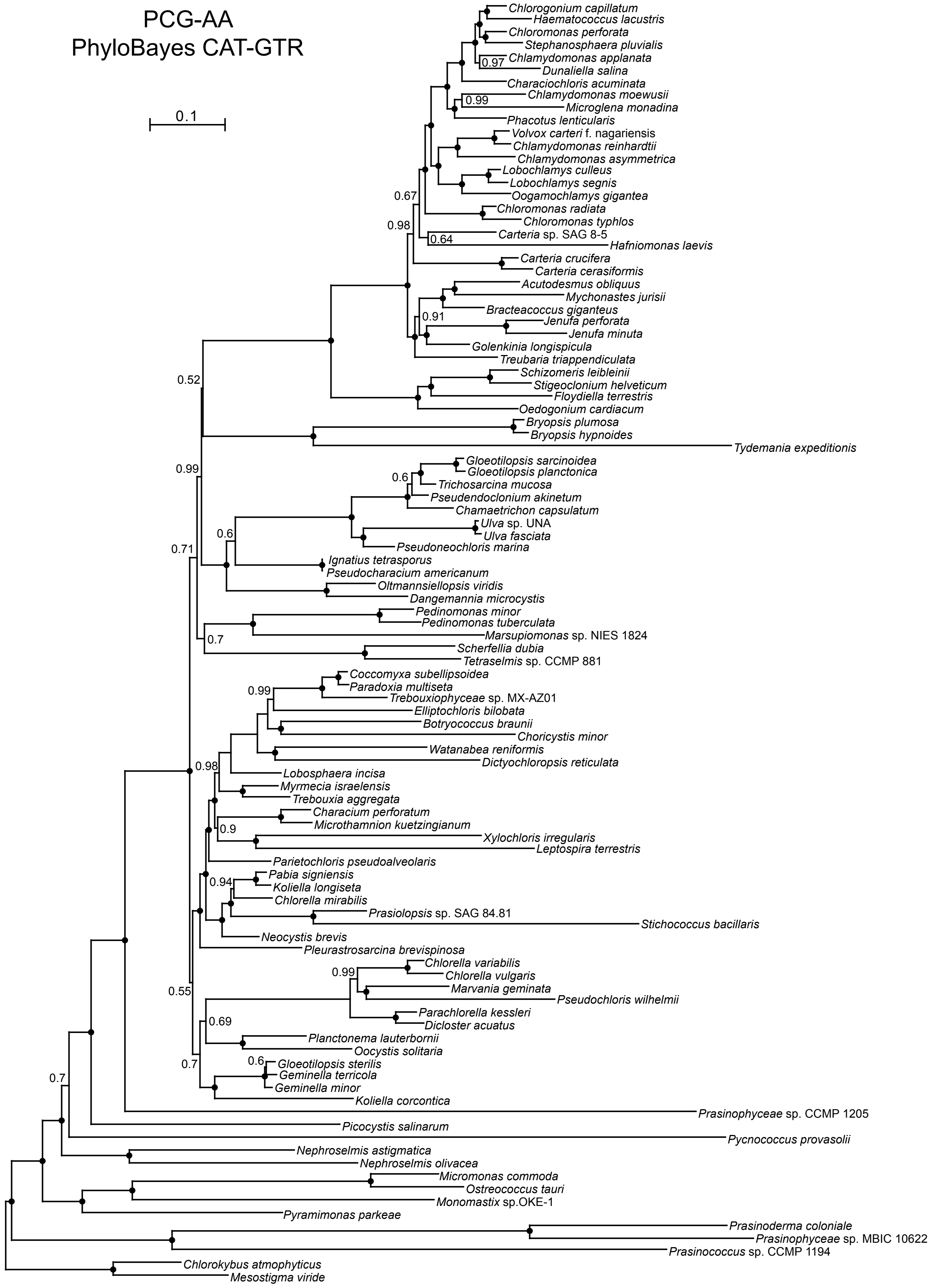
PCG-AA
PhyloBayes CAT-GTR
0.1
Chlorogonium capillatum
Haematococcus lacustris
Chloromonas perforata
Stephanosphaera pluvialis
Chlamydomonas applanata
Dunaliella salina
Characiochloris acuminata
Chlamydomonas moewusii
Microglena monadina
Phacotus lenticularis
Volvox carteri f. nagariensis
Chlamydomonas reinhardtii
Chlamydomonas asymmetrica
Lobochlamys culleus
Lobochlamys segnis
Oogamochlamys gigantea
Chloromonas radiata
Chloromonas typhlos
Carteria sp. SAG 8-5
Hafniomonas laevis
Carteria crucifera
Carteria cerasiformis
Acutodesmus obliquus
Mychonastes jurisii
Bracteacoccus giganteus
Jenufa perforata
Jenufa minuta
Golenkinia longispicula
Treubaria triappendiculata
Schizomeris leibleinii
Stigeoclonium helveticum
Floydiella terrestris
Oedogonium cardiacum
Bryopsis plumosa
Bryopsis hypnoides
Tydemania expeditionis
Gloeotilopsis sarcinoidea
Gloeotilopsis planctonica
Trichosarcina mucosa
Pseudendoclonium akinetum
Chamaetrichon capsulatum
Ulva sp. UNA
Ulva fasciata
Pseudoneochloris marina
Ignatius tetrasporus
Pseudocharacium americanum
Oltmannsiellopsis viridis
Dangemannia microcystis
Pedinomonas minor
Pedinomonas tuberculata
Marsupiomonas sp. NIES 1824
Scherffelia dubia
Tetraselmis sp. CCMP 881
Coccomyxa subellipsoidea
Paradoxia multiseta
Trebouxiophyceae sp. MX-AZ01
Elliptochloris bilobata
Botryococcus braunii
Choricystis minor
Watanabea reniformis
Dictyochloropsis reticulata
Lobosphaera incisa
Myrmecia israelensis
Trebouxia aggregata
Characium perforatum
Microthamnion kuetzingianum
Xylochloris irregularis
Leptospira terrestris
Parietochloris pseudoalveolaris
Pabia signiensis
Koliella longiseta
Chlorella mirabilis
Prasiolopsis sp. SAG 84.81
Stichococcus bacillaris
Neocystis brevis
Pleurastrosarcina brevispinosa
Chlorella variabilis
Chlorella vulgaris
Marvania geminata
Pseudochloris wilhelmii
Parachlorella kessleri
Dicloster acuatus
Planctonema lauterbornii
Oocystis solitaria
Gloeotilopsis sterilis
Geminella terricola
Geminella minor
Koliella corcontica
Prasinophyceae sp. CCMP 1205
Picocystis salinarum
Pycnococcus provasolii
Nephroselmis astigmatica
Nephroselmis olivacea
Micromonas commoda
Ostreococcus tauri
Monomastix sp.OKE-1
Pyramimonas parkeae
Prasinoderma coloniale
Prasinophyceae sp. MBIC 10622
Prasinococcus sp. CCMP 1194
Chlorokybus atmophyticus
Mesostigma viride
0.97
0.99
0.67
0.98
0.64
0.91
0.52
0.99
0.6
0.6
0.71
0.7
0.99
0.98
0.9
0.94
0.55
0.99
0.69
0.7
0.6
0.7

Figure S11

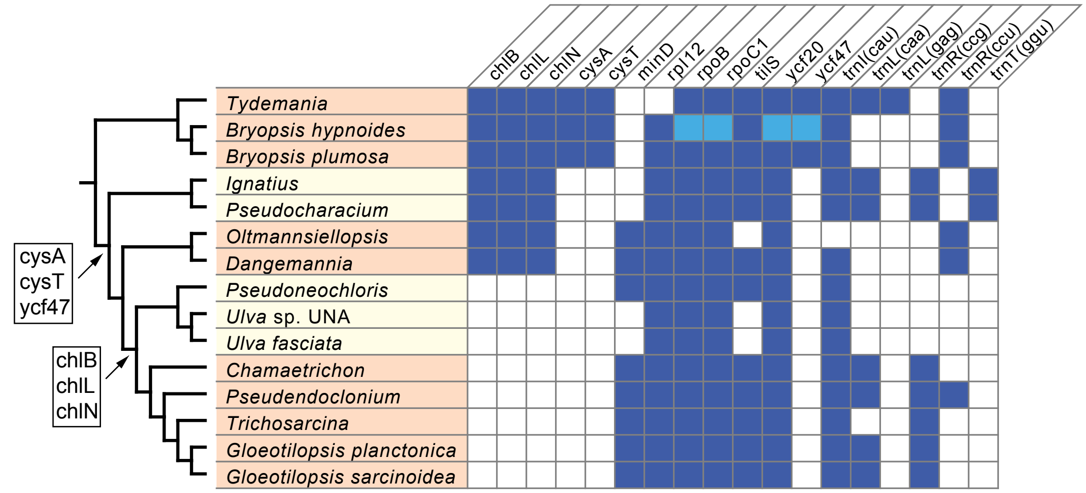
chlB
chlL
chlN
cysA
cysT
minD
rpl12
rpoB
rpoC1
tilS
ycf20
ycf47
trnI(cau)
trnL(caa)
trnL(gag)
trnR(ccg)
trnR(ccu)
trnT(ggu)
Tydemania
Bryopsis hypnoides
Bryopsis plumosa
Ignatius
Pseudocharacium
Oltmannsiellopsis
Dangemannia
Pseudoneochloris
Ulva sp. UNA
Ulva fasciata
Chamaetrichon
Pseudendoclonium
Trichosarcina
Gloeotilopsis planctonica
Gloeotilopsis sarcinoidea
cysA
cysT
ycf47
chlB
chlL
chlN

Figure S12
